# An investigation of causes of false positive single nucleotide polymorphisms using simulated reads from a small eukaryote genome

**DOI:** 10.1186/s12859-015-0801-z

**Published:** 2015-11-11

**Authors:** Antonio Ribeiro, Agnieszka Golicz, Christine Anne Hackett, Iain Milne, Gordon Stephen, David Marshall, Andrew J. Flavell, Micha Bayer

**Affiliations:** The James Hutton Institute, Invergowrie, Dundee, DD2 5DA Scotland UK; School of Agriculture and Food Sciences, University of Queensland, Brisbane, Queensland, 4072 Australia; Australian Centre for Plant Functional Genomics and School of Agriculture and Food Sciences, University of Queensland, Brisbane, Queensland, 4072 Australia; Division of Plant Sciences, University of Dundee at JHI, Invergowrie, Dundee, DD2 5DA Scotland UK; Biomathematics and Statistics Scotland, Invergowrie, Dundee, DD2 5DA Scotland UK

**Keywords:** False positive, SNP, NGS, Read mismapping, Misassembly, Mapping stringency, Read length

## Abstract

**Background:**

Single Nucleotide Polymorphisms (SNPs) are widely used molecular markers, and their use has increased massively since the inception of Next Generation Sequencing (NGS) technologies, which allow detection of large numbers of SNPs at low cost. However, both NGS data and their analysis are error-prone, which can lead to the generation of false positive (FP) SNPs. We explored the relationship between FP SNPs and seven factors involved in mapping-based variant calling — quality of the reference sequence, read length, choice of mapper and variant caller, mapping stringency and filtering of SNPs by read mapping quality and read depth. This resulted in 576 possible factor level combinations. We used error- and variant-free simulated reads to ensure that every SNP found was indeed a false positive.

**Results:**

The variation in the number of FP SNPs generated ranged from 0 to 36,621 for the 120 million base pairs (Mbp) genome. All of the experimental factors tested had statistically significant effects on the number of FP SNPs generated and there was a considerable amount of interaction between the different factors. Using a fragmented reference sequence led to a dramatic increase in the number of FP SNPs generated, as did relaxed read mapping and a lack of SNP filtering. The choice of reference assembler, mapper and variant caller also significantly affected the outcome. The effect of read length was more complex and suggests a possible interaction between mapping specificity and the potential for contributing more false positives as read length increases.

**Conclusions:**

The choice of tools and parameters involved in variant calling can have a dramatic effect on the number of FP SNPs produced, with particularly poor combinations of software and/or parameter settings yielding tens of thousands in this experiment. Between-factor interactions make simple recommendations difficult for a SNP discovery pipeline but the quality of the reference sequence is clearly of paramount importance. Our findings are also a stark reminder that it can be unwise to use the relaxed mismatch settings provided as defaults by some read mappers when reads are being mapped to a relatively unfinished reference sequence from e.g. a non-model organism in its early stages of genomic exploration.

**Electronic supplementary material:**

The online version of this article (doi:10.1186/s12859-015-0801-z) contains supplementary material, which is available to authorized users.

## Background

Single-nucleotide polymorphisms are used as molecular markers in diverse applications, such as human disease genetics, plant and animal breeding, population genetics and forensics [1–3]. The emergence of NGS technologies has yielded dramatic improvements in costs and throughput [3, 4]. However, SNP discovery in NGS data can result in significant numbers of false positives [5, 6]. In addition to sequencing errors, which vary in pattern and rate depending on the sequencing platform [7], the short read lengths that prevail in NGS, together with the repetitious nature of the genomes of many organisms, can lead to errors in the genome assembly and/or read mapping stages.

The traditional approach to SNP discovery is based on mapping reads to a reference sequence, but several new approaches have been suggested which are mapping-free (e.g. [8]). Their uptake appears to have been slow, however, and the majority of projects currently still employ a mapping-based approach for SNP discovery.

Kumar et al. (2012) have argued that SNP discovery improves with better quality reference genomes. Misassembly of the reference sequence creates the conditions required for reads to be mismapped in the first place, as the origin of a read may not be available in an imperfect assembly. This is of particular relevance for SNP discovery projects where a well assembled and well curated reference sequence is not yet available. The reference sequences of most sequenced organisms are classified as a “permanent draft” (https://gold.jgi-psf.org/statistics), and have undergone little or no manual curation following the primary assembly stage. Typically, the resulting genome sequences are fragmented and incomplete with substantial numbers of misassemblies. All of these imperfections may subsequently cause read mismapping, and our study specifically addresses the issues associated with this.

In the present work, we have investigated the effects of a number of factors on the generation of false positive SNPs (loci incorrectly identified as polymorphic) using the ∼ 125 Mbp genome of the flowering plant *Arabidopsis thaliana*. Simulated NGS read datasets varying in length from 50 to 1000 base pairs (bp) were used to generate both new genome assemblies and mappings to test the effects of NGS read length, different software for genome assembly, read mapping and SNP calling (including variable parameter settings), as well as SNP filtering, on FP SNP generation.

## Methods

### Read datasets preparation

**Fig. 1:**
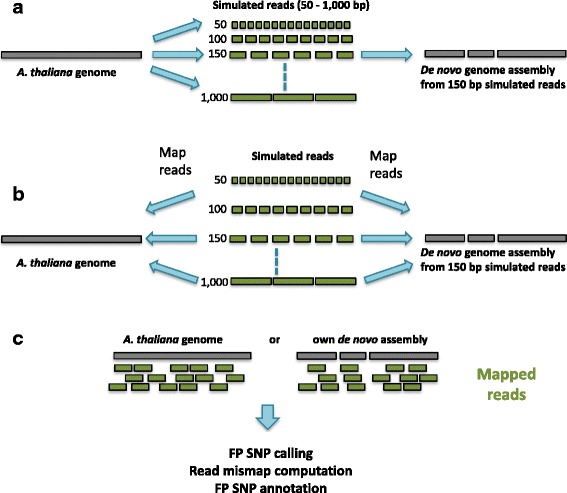
Experimental design. **a** The *A. thaliana* genome was used to generate simulated reads of different lengths. *De novo* assemblies were computed from the 150 bp read datasets using different assemblers. **b** With the assemblies as references, separate read mappings were carried out for each of the different read length datasets and with different combination of factor levels, using the original genome as a control. **c** SNP detection was carried out with different variant callers and the results were analysed to detect whether the mismatched reads causing the SNPs were due to mismapping. SNP annotation was performed to detect enrichment for particular genomic features at SNP positions

The five chromosome sequences of *Arabidopsis thaliana*, available at ftp://ftp.arabidopsis.org/home/tair/Sequences/whole_chromosomes, served as the template for the generation of the simulated reads for our study. The SimSeq read simulator (last update 4.12.2011; https://github.com/jstjohn/SimSeq) was used to generate haploid, error-free paired-end and mate-pair reads (the latter created specifically for the assembly stage) from each of the chromosome sequences (see Additional file 1: Supplementary data section SD.1). This sampling mode allowed us to assume that every SNP encountered in the mappings must be an FP SNP which is due to read mismapping as there were no other sources of variant alleles. Paired-end reads were produced with 100-fold coverage depth and at lengths of 50, 100, 150, 300, 500, and 1000 bp (Fig. 1). Fragment sizes for these were 90, 180, 270, 540, 900 and 1800 bp respectively. Mate-pair reads were produced with 50-fold coverage depth at a length of 150 bp, with a fragment size of 3000 bp. Full details of the fragment sizes are provided in Additional file 1: Supplementary data (section SD.1.1, Table S4).

### Reference genome assembly

In order to provide the conditions typical of a non-model organism use case, two reference sequences for the read mapping were *de novo* assembled from the 150 bp read datasets, one using the Velvet assembler version 1.2.10 [9] and the other using the Allpaths-LG assembler version r51511 [10, 11].

To keep the design of the experiment simple, we used only the 150 bp read datasets for assembly. The depth of coverage for the assemblies was 150x, where 100x was contributed by the 150 bp paired-end reads dataset, while 50x was contributed by the mate-pair reads. Each assembler was run twice, using separately simulated read datasets. Additional information about the assembly process can be found in the Additional file 1: Supplementary data (section SD.2).

To assess the degree of difference between the *de novo* assembled reference sequence and the *A. thaliana* genome sequence (the control for the read mapping), we analysed each replicate assembly with QUAST [12], using the *A. thaliana* genome sequence and the gene models as the benchmark dataset. The results from this are shown in the Additional file 1: Supplementary data (section SD.2; Table S5). Definitions of the metrics employed by QUAST are available in the online manual for this software (http://quast.bioinf.spbau.ru/manual.html#sec3.1.1).

### Read mapping

Each of the six read datasets (50–1000 bp) was mapped to the *de novo* assemblies and the *A. thaliana* control (see below) with Bowtie2 version 2.2.1 [13] and BWA-SW version 0.7.10-r789 [14], both widely used alignment tools [5] capable of dealing with the range of read lengths explored in the study. In order to keep coverage comparable among all mappings, we used the same mismatch rate across all read lengths, rather than a fixed number of mismatches. To enable any SNPs to be called, at least one mismatch per read must be allowed. With a minimum read length of 50 bp this equates to a mismatch rate of 1 mismatch in 50 bp, or 2 %. We aimed to compare strict and relaxed mismatch stringencies, and thus we chose the default of the latest BWA algorithm as the relaxed setting. This was calculated as being equivalent to 14 % mismatches per read. We then applied both mismatch rates (2 % and 14 %) to each of the mappers. Additional file 1: Supplementary data section SD.3 describes how the parameter settings were calculated for each mapper.

### SNP calling

The FreeBayes variant caller (version v0.9.18-3-gb72a21b, https://github.com/ekg/freebayes, [15]) and the Genome Analysis Toolkit (GATK, version 3.3-0, https://www.broadinstitute.org/gatk/, [16, 17]) were run over each of the mappings separately. Both tools were chosen for SNP discovery as they are widely used [18] and provide substantial configurability.

To speed up the SNP calling in FreeBayes, we produced a Java SE 7/SAMtools 0.1.18 [19] wrapper around it that splits and parallelises the job across multiple nodes and processors of a compute cluster. This allowed the jobs to run in a fraction of the time that would otherwise have been required. This is achieved by querying the list of contigs, discarding those that have no reads mapped to them, splitting the remainder into discrete regions that can be processed independently by FreeBayes, before finally concatenating the results back together into a single VCF (Variant Call Format) file.

For GATK, we designed a pipeline script to perform duplicate markup with Picard Tools (version 1.119 (http://broadinstitute.github.io/picard)), and local realignment around indels and variant calling with GATK. The base quality recalibration step was left out as we did not have known variants as part of our study design. To evaluate the effect of the mapping quality, both variant callers were configured to run with (MAPQ = 20) and without (MAPQ = 0) mapping quality filtering. The detailed parameters used in FreeBayes and GATK are available in the Additional file 1: Supplementary data section SD.4.

We also included filtering of SNPs by read depth as an additional experimental factor (maximum read depth 150 *versus* no filtering). Depth filtering can be applied to remove SNPs located in large accumulations of reads in regions that e.g. represent collapsed repeats in the reference sequence and consequently attract large numbers of reads.

In order to provide more realistic final SNP numbers, we also removed multiallelic SNPs from all resulting VCF files, as well as SNPs with SNP quality scores of less than 20.

### Control dataset

**Fig. 2:**
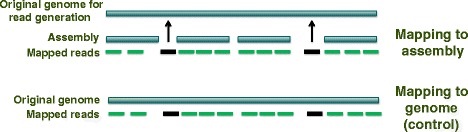
Control conceptualized. The reads indicated by arrows cannot be mapped to their original positions in the *de novo* reference genome assembly (Section “Reference genome assembly”), due to gaps or misassembly and reads may therefore map to the wrong location, which potentially results in FP SNPs. In the control mapping to the complete genome, the same reads can map back correctly to their original positions

The five chromosome sequences of *A. thaliana* (see section “Read datasets preparation”) were combined to be used as a control reference sequence for the study. The read mapping and SNP calling stages were also applied to this original genome sequence. Using the original reference should theoretically yield no or at least fewer SNPs as the additional complication of the *de novo* assembly is removed here, and can therefore be used as a control for the *de novo* assembled reference sequences. Figure 2 illustrates the concept of the control.

### Read mismapping quantification stage

A custom pipeline consisting of our own Java code, and other resources including the Picard API (http://sourceforge.net/projects/picard/), SAMtools version 0.1.18 [19], and BLASTN [20], was used to quantify instances where mismapped reads caused SNPs, taking advantage of the read origin information generated by the read simulator (Additional file 1: Supplementary data section SD.5; for source code availability see SD.13). For each SNP, the code quantified the percentage of unique overlapping (covering) reads which contained the alternate allele and originally belonged to a different chromosome or different region in the same chromosome, indicating mismapping of reads. To avoid redundancy, only those SNPs were considered that had not been filtered out by the depth filter. The Additional file 1: Supplementary data section SD.6 shows the code workflow in detail.

### SNP annotation

We tested whether the regions containing SNPs were enriched for a given type of genomic feature, such as intergenic regions, gene families, pseudogenes, repeats, transposons, etc. We also compared the proportions of features observed in the FP SNPs with those for the entire genome. SNP manifests (SNP site plus approximately 120 bp flanking region either side) were extracted from the *de novo* assembly sequence and BLASTed against a database composed of coding sequences (CDS) and intergenic regions (ftp://ftp.arabidopsis.org/home/tair/Sequences/blast_datasets/TAIR10_blastsets/) retrieved from the *A. thaliana* annotation. The same procedure was performed on the control mapping. The steps required to build the BLAST database are detailed in the Additional file 1: Supplementary data section SD.7.

### Replicate workflow runs

**Fig. 3:**
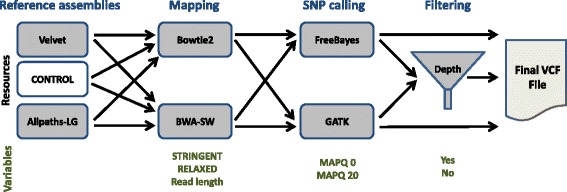
Tools and variables used in the experiment. Paired-end datasets of differing read lengths (50–1,000 bp) were mapped using Bowtie2 and BWA-SW with either high (2 % mismatches) or low (14 % mismatches) mapping stringency. The *de novo* assemblies computed with Velvet and Allpaths-LG were used as references, as well as the original *A. thaliana* reference sequence (control). All the resulting mappings underwent SNP calling with the variant callers FreeBayes and GATK, with and without filtering for read mapping quality. The resulting SNPs were filtered by coverage depth (< 150) and these call sets were compared to their unfiltered counterparts. For the final SNP counts, only biallelic entries with a SNP quality score greater than 20 were used

To ensure reproducibility and consistency, the experiment was carried out in duplicate. For each read length, two independent, randomly sampled read sets were created, and a new assembly was made from the 150 bp read datasets using both Velvet and Allpaths-LG. The mapping of all read datasets, SNP calling, and the SNP annotation were performed with both the *de novo* assemblies and the whole genome control as reference sequences for each factor combination. Additional information about the replicate assemblies is also available in the Additional file 1: Supplementary data (section SD.2; Table S5). Figures 1, 2 and 3 summarise the study’s experimental design and the application of tools and variables.

### Statistical analysis

Analysis of variance (ANOVA) was used to test for significant effects of the seven factors and all possible interactions on the number of false positives detected. The replicate effect was fitted as a random effect, while all other effects and interactions were fitted as fixed effects. The untransformed number of false positives did not satisfy the usual ANOVA assumptions of normally distributed residuals with constant variance. The number of FP SNPs was therefore analysed after a *log*_10_(N+1) transformation, which improved the distribution of the residuals. A random permutation test with 999 permutations was also run to obtain a non-parametric estimate of the significances of each effect, and this gave very similar probabilities to the usual ANOVA F probabilities. The analysis was carried out using GenStat 16 for Windows [21].

## Results

Our strategy for exploring the origins of FP SNP generation is shown in Fig. 1. Sets of simulated reads of varying sizes were sampled from the *A. thaliana* genome sequence. To explore the effect of assembly on FP SNP generation, two different reference sequences were generated using the *de novo* assemblers Velvet and Allpaths-LG. To simplify the design of the experiment, we used only the 150 bp read length dataset for assembly. Our choice of this read length was based on two considerations: a) a large number of ongoing sequencing projects use Illumina Hiseq reads as their primary source of sequence and the current maximum read length for this is 150 bp (http://systems.illumina.com/systems/hiseq_2500_1500/performance_specifications.ilmn), and b) even projects involving the assembly of very large, complex genomes such as wheat [22] use reads as short as this or even shorter (barley [23], norway spruce [24]) as their primary source of sequence.

To investigate variations in read mapping, the simulated read sets described above were then mapped to the two *de novo* genome assemblies, as well as the *A. thaliana* reference genome, using two widely used read mappers, Bowtie2 and BWA. The range of read lengths chosen covers most of the currently available sequencing technologies, with the exception of Pacific BioSciences and Oxford Nanopore ([25], and updates at http://www.molecularecologist.com/next-gen-fieldguide-2014/). The latter two technologies produce longer reads but are currently associated with substantial error rates and their use in variant calling is still in its early stages. The mappings generated were then processed with two popular variant callers, GATK and FreeBayes.

### General observations

The range of FP SNP numbers observed in the experiment varied from 0 to 36,621, depending upon the choice of reference sequence, tools and parameters. Out of 576 factor level combinations, 211 contained zero FPs (Additional file 1: Supplementary file snpNumbersStats.xlsx). These included sets using the BWA mapper on the “strict” mismatch setting with the GATK variant caller for all combinations of depth filtering/no depth filtering, all three assembly types, MAPQ settings of 0 or 20, and the full range of read lengths. Zero FP SNPs were also found for sets using the BWA mapper on the “strict” mismatch setting with the FreeBayes variant caller and a MAPQ setting of 20 for all combinations of depth filtering/no depth filtering, all three assembly types, and the full range of read lengths. For the control assembly only, the FP count remained at zero in the combinations above even if the “relaxed” mismatch setting was used. The Bowtie2 mapper found zero FPs for the control assembly only and read lengths of 150 bp or fewer, with all combinations of depth filtering/no depth filtering, variant caller, stringency and MAPQ settings, as well as on the “strict” setting with 500 or 1000 bp reads. None of the mappings against the *de novo* assemblies achieved a zero FP count on the relaxed mismatch setting. At the other end of the spectrum, the largest mean number of FPs encountered was 36,260.5 (300 bp reads, Allpaths assembly, relaxed Bowtie2 mapping, MAPQ filter 0, FreeBayes, no depth filtering).

The majority of factor level combinations in the control group (139 out of 192) contained no FP SNPs at all, and most of the remainder had less than 1000 FP SNPs (Additional file 1: Supplementary file snpNumbersStats.xlsx). There was, however, a large amount of variability within the control group, and some call sets contained very large numbers of FP SNPs. The worst performing combination in the control group comprised 300 bp reads mapped with Bowtie2 using relaxed mapping, variant-called with FreeBayes, using no depth filter and a MAPQ filter of 0, and yielded an average of 20,471.5 FP SNPs. The equivalent combination of tools using the strict mapping setting resulted in an average of only 17.0 FPs, a reduction of 3 orders of magnitude. This is a powerful illustration of the drastic effect of mapping stringency on FP SNP discovery.

### Main effects and interactions among experimental factors

**Fig. 4:**
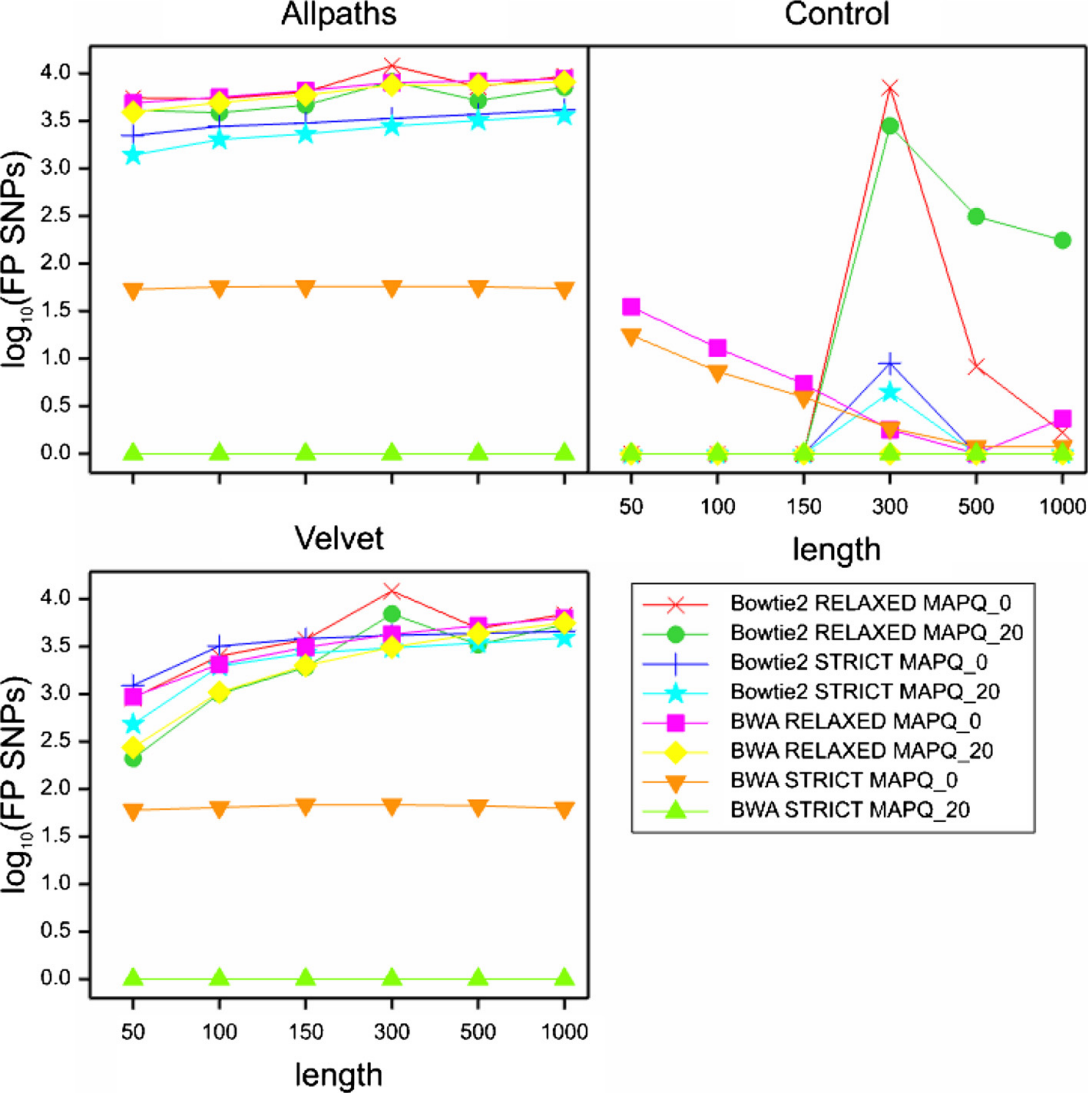
5-way interaction between assembly, mapper, read length, MAPQ, and mapping stringency. Trellis plots for the first major higher-order interaction that summarise most of the variability attributed to interaction terms

**Fig. 5:**
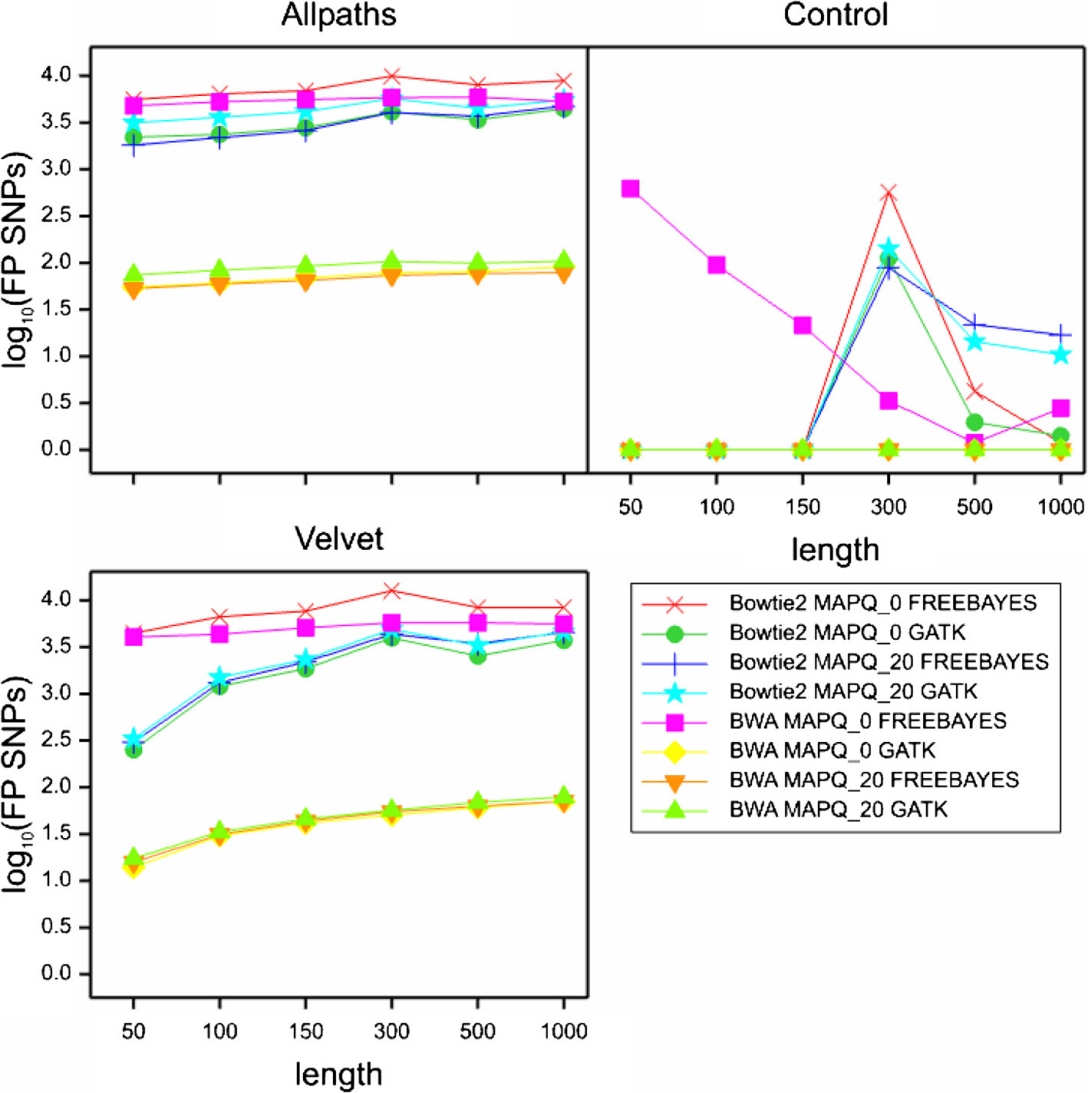
5-way interaction between assembly, mapper, variant caller, MAPQ, and read length. Trellis plots for the second major higher-order interaction that summarise most of the variability attributed to interaction terms

All factors, apart from experimental replicate, had highly significant main effects on FP SNP number in the multifactorial ANOVA (Table 1 and Additional file 1: Supplementary file ANOVA_FullResults.xlsx). However, there was a large number of highly significant higher-order interaction terms in the ANOVA results, and these indicated many complex interactions between experimental factors. The results presented here should be viewed in the context of these interactions, as global means hide much of the complexity of our findings. Figures 4 and 5 show trellis plots for the two major higher-order interactions that summarise most of the variability attributed to interaction terms. The equivalent numerical values are shown in Tables 2 and 3. The residual term due to differences among the replicates accounted for less than 0.03 % of the total variation.

**Table 1:** Main effects from the factorial Analysis of Variance (ANOVA). For the full list of all possible interaction terms please see the Additional file 1: Supplementary file ANOVA_FullResults.xlsx (the residual term here is from the full ANOVA)

Source of variation	d.f.	s.s.	m.s.	v.r.	F prob.	perm prob.	Percentage SS
Replicate stratum	1	0.01693	0.01693	8.82			
Replicate.*Units* stratum							
Length	5	40.79358	8.15872	4247.34	0.000	0.001	1.18
Assembly	2	1516.31545	758.15772	394688.33	0.000	0.001	43.90
Mapper	1	265.90685	265.90685	138428.09	0.000	0.001	7.70
Stringency	1	371.94519	371.94519	193630.46	0.000	0.001	10.77
MAPQ	1	55.69223	55.69223	28992.74	0.000	0.001	1.61
Variant caller	1	73.45412	73.45412	38239.38	0.000	0.001	2.13
Depth filter	1	5.92562	5.92562	3084.81	0.000	0.001	0.17
Residual	575	1.10452	0.00192

**Table 2:** First major higher-order interaction. *L* *o* *g*_10_-transformed means for the 5-way interaction between assembly, mapper, read length, MAPQ, and mapping stringency

			Length	50	100	150	300	500	1000
Assembly	Mapper	Stringency	MAPQ						
Allpaths	Bowtie2	Relaxed	0	3.743	3.736	3.807	4.084	3.863	3.975
Allpaths	BWA	Relaxed	0	3.692	3.752	3.823	3.906	3.922	3.947
Allpaths	Bowtie2	Strict	0	3.349	3.445	3.480	3.529	3.571	3.621
Allpaths	BWA	Strict	0	1.729	1.755	1.760	1.759	1.758	1.739
Control	Bowtie2	Relaxed	0	0.000	0.000	0.000	3.856	0.920	0.226
Control	BWA	Relaxed	0	1.547	1.112	0.736	0.254	0.000	0.369
Control	Bowtie2	Strict	0	0.000	0.000	0.000	0.953	0.000	0.000
Control	BWA	Strict	0	1.248	0.866	0.595	0.270	0.075	0.075
Velvet	Bowtie2	Relaxed	0	2.960	3.399	3.572	4.084	3.691	3.838
Velvet	BWA	Relaxed	0	2.972	3.313	3.491	3.628	3.720	3.799
Velvet	Bowtie2	Strict	0	3.091	3.504	3.582	3.618	3.638	3.658
Velvet	BWA	Strict	0	1.779	1.806	1.834	1.834	1.825	1.798
Allpaths	Bowtie2	Relaxed	20	3.615	3.589	3.668	3.917	3.716	3.857
Allpaths	BWA	Relaxed	20	3.594	3.695	3.775	3.880	3.882	3.914
Allpaths	Bowtie2	Strict	20	3.143	3.306	3.366	3.448	3.507	3.562
Allpaths	BWA	Strict	20	0.000	0.000	0.000	0.000	0.000	0.000
Control	Bowtie2	Relaxed	20	0.000	0.000	0.000	3.451	2.497	2.246
Control	BWA	Relaxed	20	0.000	0.000	0.000	0.000	0.000	0.000
Control	Bowtie2	Strict	20	0.000	0.000	0.000	0.648	0.000	0.000
Control	BWA	Strict	20	0.000	0.000	0.000	0.000	0.000	0.000
Velvet	Bowtie2	Relaxed	20	2.322	3.002	3.281	3.842	3.519	3.733
Velvet	BWA	Relaxed	20	2.438	3.018	3.300	3.492	3.637	3.748
Velvet	Bowtie2	Strict	20	2.682	3.292	3.429	3.486	3.536	3.590
Velvet	BWA	Strict	20	0.000	0.000	0.000	0.000	0.000	0.000
Sed = 0.02191									

*Sed* standard error of the difference

**Table 3:** Second major higher-order interaction. *L* *o* *g*_10_-transformed means for the 5-way interaction between assembly, mapper, variant caller, MAPQ and read length

			Length	50	100	150	300	500	1000
Assembly	Mapper	Variant caller	MAPQ						
Allpaths	Bowtie2	FreeBayes	0	3.75	3.81	3.84	4.00	3.90	3.95
Allpaths	BWA	FreeBayes	0	3.68	3.72	3.75	3.77	3.77	3.73
Allpaths	Bowtie2	GATK	0	3.34	3.37	3.45	3.61	3.53	3.65
Allpaths	BWA	GATK	0	1.74	1.78	1.84	1.90	1.91	1.96
Control	Bowtie2	FreeBayes	0	0.00	0.00	0.00	2.76	0.63	0.08
Control	BWA	FreeBayes	0	2.80	1.98	1.33	0.52	0.08	0.44
Control	Bowtie2	GATK	0	0.00	0.00	0.00	2.05	0.29	0.15
Control	BWA	GATK	0	0.00	0.00	0.00	0.00	0.00	0.00
Velvet	Bowtie2	FreeBayes	0	3.65	3.82	3.88	4.10	3.92	3.92
Velvet	BWA	FreeBayes	0	3.61	3.64	3.71	3.76	3.76	3.75
Velvet	Bowtie2	GATK	0	2.40	3.08	3.27	3.60	3.41	3.57
Velvet	BWA	GATK	0	1.14	1.48	1.62	1.70	1.78	1.85
Allpaths	Bowtie2	FreeBayes	20	3.26	3.34	3.42	3.61	3.57	3.68
Allpaths	BWA	FreeBayes	20	1.72	1.77	1.81	1.87	1.89	1.90
Allpaths	Bowtie2	GATK	20	3.50	3.56	3.62	3.76	3.65	3.74
Allpaths	BWA	GATK	20	1.87	1.92	1.96	2.01	2.00	2.02
Control	Bowtie2	FreeBayes	20	0.00	0.00	0.00	1.95	1.34	1.23
Control	BWA	FreeBayes	20	0.00	0.00	0.00	0.00	0.00	0.00
Control	Bowtie2	GATK	20	0.00	0.00	0.00	2.15	1.16	1.02
Control	BWA	GATK	20	0.00	0.00	0.00	0.00	0.00	0.00
Velvet	Bowtie2	FreeBayes	20	2.48	3.12	3.34	3.64	3.54	3.66
Velvet	BWA	FreeBayes	20	1.20	1.50	1.64	1.74	1.80	1.85
Velvet	Bowtie2	GATK	20	2.52	3.17	3.37	3.69	3.52	3.67
Velvet	BWA	GATK	20	1.24	1.52	1.66	1.75	1.84	1.90
Sed = 0.02191									

#### Assembly

The reference sequence used had the most pronounced effect on the rate of FP SNPs, accounting for 43.9 % of the total variation in the data (Table 1), with a highly significant main effect. There were significant interactions with all six of the other factors. Mappings against the original *A. thaliana* genome (Control) yielded comparatively few FP SNPs in most cases (Figs. 4 and 5), while mappings against our own *de novo* assemblies generally produced FP SNP numbers orders of magnitude greater, with the Velvet reference sequence outperforming the Allpaths sequence slightly in most cases.

#### Stringency

Mapping stringency accounted for 10.8 % of the total variation in the data, making it the second most important factor in the experiment (Table 1). The main effect in the ANOVA was statistically highly significant, with the global means suggesting a reduction of approximately one order of magnitude in FP numbers for the “strict” setting (*log*_10_-transformed means: relaxed 2.64; strict 1.50). This effect was observable in the majority of interactions analysed here (Tables 2, 4, 5, and Figs. 4 and 5). The reduction in FP numbers from applying the strict mismatch setting was greatest for the combination of BWA and the two poorer reference sequences, and for the combination of Bowtie2 and the Control reference sequence with read lengths of 300–1000 bp.

**Table 4:** Mapper and mapping stringency interaction. *L* *o* *g*_10_-transformed means for the interaction between mapper and mapping stringency

	Stringency	Relaxed	Strict
Mapper			
Bowtie2		2.7780	2.3343
BWA-SW		2.5099	0.6807
Sed = 0.00365

**Table 5:** Assembly type, mapper, and mapping stringency interaction. *L* *o* *g*_10_-transformed means for the interaction between assembly type, mapper and mapping stringency

	Mapper	Bowtie2		BWA-SW	
	Stringency	Relaxed	Strict	Relaxed	Strict
Assembly					
Allpaths-LG		3.7976	3.4439	3.8151	0.8750
Control		1.0996	0.1334	0.3349	0.2608
Velvet		3.4369	3.4256	3.3796	0.9063
Sed = 0.00633					

#### Mapping tools

This was the third most important factor in FP SNP generation, in terms of the contribution to the overall variation in the data, contributing 7.7 % of the total (Table 1). On average, BWA produced fewer FPs than Bowtie2 (*log*_10_ transformed means: 1.59 *vs* 2.55 respectively) but deviations from this pattern occurred depending on the read length, MAPQ, mapping stringency and reference sequence (Tables 2 and 4; Figs. 4 and 5). Most of these occurred in the relaxed mappings with MAPQ_0 filtering. For the short read mappings (50–150 bp) against the Control reference with MAPQ_20 filtering, both mappers performed equally well. However, even on the most conservative settings (strict mapping, MAPQ_20) and with the best reference sequence (Control), Bowtie2 performed poorly on the 300 bp reads, whereas on the longer reads (500/1000 bp) its performance matched that of BWA (Table 2).

#### Variant caller

The effect of the variant calling software, again, was statistically highly significant but had interdependencies with other factors. Global means suggested that GATK produced fewer FPs than FreeBayes but this only held true for the MAPQ_0 call sets. When a MAPQ filter of 20 was applied the GATK FP rates in most cases were either equal to or slightly higher than those obtained with FreeBayes (Tables 3 and 6).

**Table 6:** MAPQ and variant caller interaction. *L* *o* *g*_10_-transformed means for the interaction between MAPQ filter level and variant caller

	Variant caller	FreeBayes	GATK
MAPQ			
0		2.8277	1.7635
20		1.8287	1.8830
Sed = 0.00365			

#### MAPQ based filtering of SNPs

Read mapping quality based filtering of SNPs (0 *versus* 20) also had a significant main effect, and while the global means suggested that MAPQ filtering of SNPs reduces FP numbers (*log*_10_ means: MAPQ_0 = 2.29; MAPQ_20 = 1.85), this did not apply universally. When filtering for MAPQ_20, FP numbers were reduced for the FreeBayes call sets but not for GATK call sets (Table 6).

#### Read length

FP SNP numbers did not strictly decrease as a function of read length (Figs. 4 and 5). This contradicts the assumption that longer reads lead to fewer FP SNPs due to higher mapping accuracy. Instead, FP SNP numbers in most call sets were either flat when plotted against read length, or showed an asymptotic increase with read length. Only the BWA/MAPQ_0 call sets in the Control group showed a decline of FP numbers with read length, with a minimum at 500 bp and a slight increase at 1000 bp. In the Control group only, the Bowtie2 mappings had a sharp peak in FP numbers for read length 300 bp, with the 500 bp and 1000 bp FP numbers still higher than those for the shorter reads (50–150 bp), all of which had zero FPs regardless of any other factors.

#### Depth filter

Filtering SNPs for read depth greater than 150x coverage resulted in lower FP numbers, and the main effect for this was statistically highly significant (Table 1). The magnitude of this effect depended on the quality of the reference though, as shown in Table 7. The effect of applying depth filtering was strong for the two *de novo* assemblies but relatively small for the Control mappings against the intact *A. thaliana* genome.

**Table 7:** Assembly and depth filter interaction. *L* *o* *g*_10_-transformed means for the interaction between assembly and depth filter

Depth filter	No	Yes
Assembly		
Allpaths	3.1273	2.8385
Control	0.4634	0.4509
Velvet	2.8516	2.7226
Sed = 0.00447		

### Read mismapping statistics, SNP annotation and genomic distribution of FP SNP sites

**Fig. 6:**
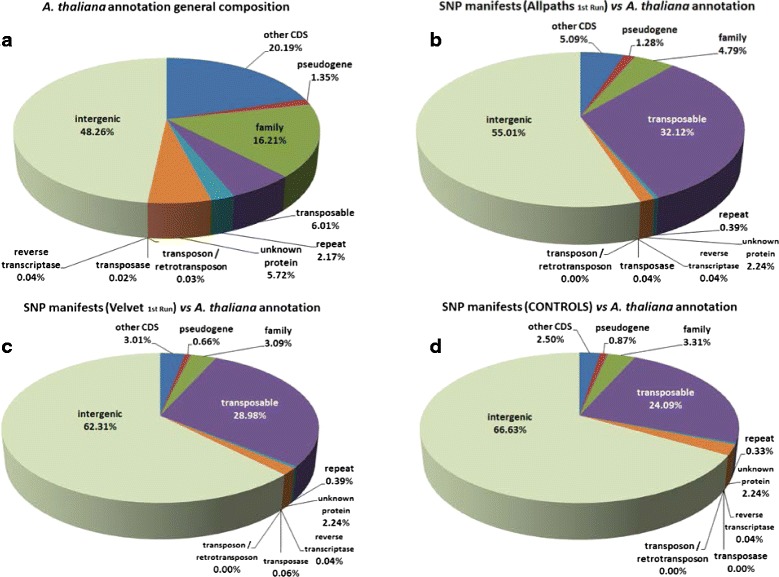
SNP annotation. **a** General composition of the *Arabidopsis thaliana* annotation compared with the BLAST-based annotation results for the SNP manifests from the first run replicates of (**b**) Allpaths-LG, (**c**) Velvet, and (**d**) the control runs (compiled)

The proportion of mismapped reads among reads with alternate alleles at SNP locations was approximately 89 % when averaged across all mappings containing FP SNPs (Additional file 1: Supplementary data section SD.10). Regions associated with FP SNPs were significantly enriched for transposable element sequences (approximately 30 %) (Fig. 6; Additional file 1: Supplementary data section SD.11), compared to approximately 6 % in the whole genome annotation.

**Fig. 7:**
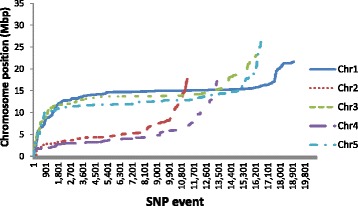
SNP locations. Plot of SNP locations by chromosome from the first Velvet assembly replicate (see Additional file 1: Supplementary data section SD.12 for data from other runs). SNP events on the y axis are ordered by their position on the chromosome

The distributions of the FP SNPs on the five *A. thaliana* chromosomes are shown in Fig. 7. The great majority of FP SNPs are found in the central (pericentromeric) regions of chromosomes. The pericentromeric regions contain high concentrations of repetitive transposable elements [26], suggesting that FP SNP generation is predominantly associated with the inability of genome assemblers and read mappers to cope with highly repetitious genome sequences.

## Discussion

### Role of the reference sequence

One of the main factors we aimed to explore here was the role of the reference sequence in FP SNP generation and how reference sequence quality affects read mismapping and consequent FP SNP accumulation. We therefore mapped reads against the published genome of *A. thaliana*, as well as *de novo* assemblies of our simulated NGS reads. The publicly available genome of *A. thaliana* has been sequenced with Sanger technology [26] and has undergone many years of labour-intensive manual curation. This is in stark contrast to the reference sequences for many non-model organisms which may be the product of relatively limited sequencing, minimal assembly effort and little subsequent quality control or validation. In this scenario, significant swathes of the genome may be misassembled or not assembled at all, and consequently read mismapping may occur on a large scale because the true targets for reads are not available in many cases. This can lead to mismatches with the reference sequence which produce FP SNPs that look inconspicuous in every respect and are therefore difficult to remove by filtering.

The difference in FP SNP numbers brought on by providing our own *de novo* assembled reference amounted to several thousands as a result of misassembly or non-assembly alone. The genome used here is small (approx. 125 Mbp) and contains relatively few repeats [26]. The effects observed here (and consequently false positive numbers) are likely to be much more pronounced with larger, more complex genomes where misassembly is much more prevalent. Large, complex genomes of this kind are common in plants [27] and other organisms.

We also observed significant numbers of FP SNPs in some of the control call sets based on mapping against the *A. thaliana* sequence. This was surprising, but seemed to be mostly due to certain unfavourable combinations of tools and parameters. The majority of call sets in the controls (282 out of 384) contained no FP SNPs at all, and most of the remainder had less than 1000 FP SNPs. All of the control call sets with more than 1000 FP SNPs (*n* = 20) were done on the relaxed mapping settings which brings home the importance of conservative mapping even when the reference sequence is well assembled.

### Choice of tools for assembly, mapping and variant calling

This study did not aim to compare the performance of specific tools involved in variant calling, but rather to provide proof of principle that false discovery rates in SNP calling can be significantly affected by the quality of reference sequence, tool choice and tool parameters. Equally, the current study did not aim to explore whether longer reads, or indeed longer read fragments, provide better *de novo* assemblies, as this has been covered elsewhere [28, 29].

The assembly tools used for producing our *de novo* reference sequences from the simulated reads comprised Velvet and Allpaths-LG. Velvet is one of the first generation of short read assemblers but has had continuous improvements and updates over many years [9, 30]. Allpaths-LG is a relatively recent tool and developers have taken a new approach by requiring input of at least two different fragment size libraries to ensure a high quality assembly. Allpaths consistently performed well in both of the Assemblathon competitions [31, 32], so we were surprised that the reference sequence produced by this tool was inferior to that produced by Velvet for most of the major metrics in the QUAST analysis (N50, assembly length, # misassemblies, genome fraction, # genes, largest contig), and it consistently yielded greater numbers of FP SNPs than the corresponding Velvet assemblies.

The two mapping tools used here, Bowtie2 and BWA, are arguably among the most commonly used tools for short read mapping. Both provide a good trade-off between accuracy and performance [33, 34] and have stood the test of time probably for this reason. On average, BWA performed better in this study, but when mapping short (50–150 bp) reads against the good quality Control reference sequence with MAPQ_20 filtering, both tools performed equally well, giving zero false positives.

### SNP filtering

Filtering by MAPQ and maximum read depth both cut FP SNP numbers significantly. Their contribution to the overall variation in the data was relatively small but it is very clear from the data that these filters should be applied wherever it is appropriate. A notable exception for this is data where large differences in read coverage are expected, for example RNAseq — here, a depth filter would be counterproductive. The effect of MAPQ filtering was less clear-cut — applying the MAPQ_20 filter to the GATK callsets actually increased FP numbers slightly in this experiment. This is counterintuitive and requires further investigation. For the FreeBayes call sets, FP numbers did drop when the MAPQ_20 filter was applied, and it is clear from these results that this should be applied as a matter of routine when using this variant caller.

### Read length

**Fig. 8:**
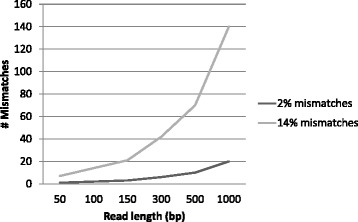
Mismatches *versus* read length. Numbers of theoretically possible mismatches per read as a function of read length and mismatch settings

The numbers of FP SNPs observed as a function of read length ran counter to our prior expectation that longer reads should result in fewer FP SNPs due to greater mapping specificity and therefore reduced mismapping rates. We only observed this for the two MAPQ_0 BWA mappings against the Control reference sequence. For most of the other call sets, FP SNP numbers increased with read length. In the Bowtie2 mappings against the Control reference sequence, the pattern observed had a sharp peak for the 300 bp read mappings. The potential to cause FP SNPs seems to be related to the length of the read, providing that reads are mapped with the same mismatch *rate* as length increases, as was the case in our experiment. Every mismatch with the reference has the potential to become a FP SNP if suitable numbers of reads are mismapped together, and both longer reads and greater mismatch rates exacerbate this problem in theory (Fig. 8).

**Fig. 9:**
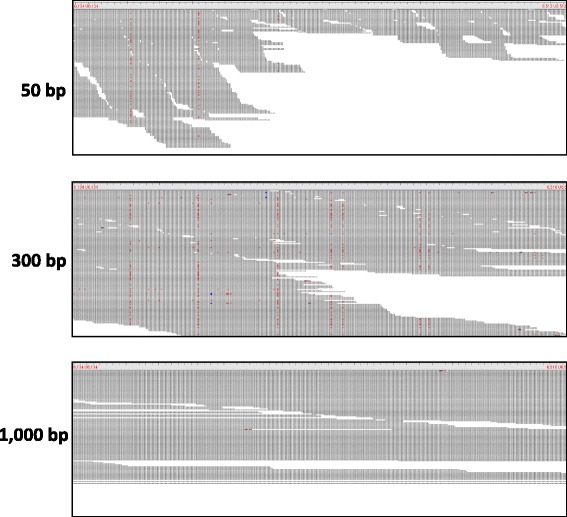
Tablet screenshots of read mismapping and ensuing FP SNPs. All screenshots show the same region on chromosome 1, which has been mapped with reads from the correct region on chromosome 1, but also reads from chromosome 2. FP SNPs are visible as vertical, red dotted lines. In this example, the 50 bp reads (*top*) introduce a small number of FP SNPs, the 300 bp (*middle*) reads introduce a substantially larger number, but in the mapping of the 1,000 bp reads (*bottom*) there are no FP SNPs, presumably indicating that the 1,000 bp reads from the contaminating region on chromosome 2 contain too many mismatches to be mapped here

This is also illustrated by the example shown in Fig. 9. Here, Tablet [35, 36] screenshots are shown of the same region in mappings of different read lengths (only 50, 300 and 1000 bp shown for brevity) for what is otherwise the same factor level combination (Allpaths reference sequence, relaxed Bowtie2 mapping). This is a region that is clearly prone to read mismapping and it would appear from inspection of the 50 and 300 bp mappings alone that the longer the reads, the more FP SNPs are generated. However, the 1000 bp read mapping shows no signs of SNPs, and it appears as though the 1000 bp reads from the region that contributes the crossmapped reads in the 50 and 300 bp mappings simply have too many mismatches to be mapped here. This suggests that greater mapping specificity does play a role in this example, and for this particular region the use of longer reads has prevented mismapping and the ensuing FP SNPs. Visual inspection of our data has produced many other examples where the 1000 bp mapping instead contained even larger numbers of FP SNPs than any of the comparable shorter read mappings, but also cases where the 50 bp mapping was the only one containing any FP SNPs at all. Taken together, this is indicative of local variation in the potential for longer reads having greater mapping specificity — whether or not read length makes a difference clearly depends on the underlying sequence context, and this will have contributed a significant amount of noise to our data which is obvious from both the plots and the data analysis.

**Fig. 10:**
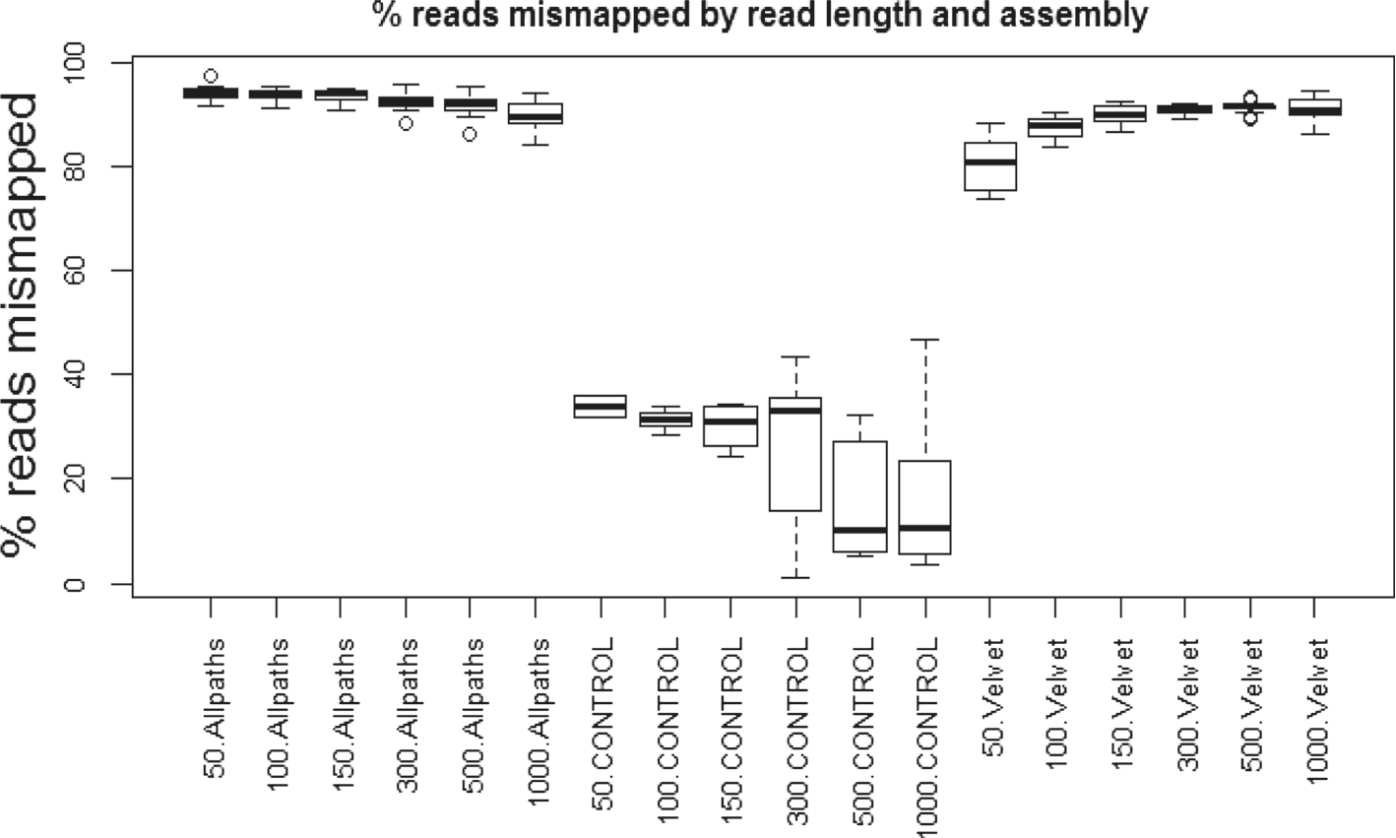
Percentages of mismapped reads as a function of read length and type of reference assembly. Mismapped reads were defined as reads at SNP locations that contained the alternate allele and originated from a different chromosome or a different region on the same chromosome. Boxplots show means (*thick black horizontal bar*), 25^th^ and 75^th^ centiles (*ends of rectangles*), 10^th^ and 90^th^ centiles (whiskers) plus individual outliers (*circles*)

The potential of the longer reads to cause greater damage seems to be mitigated at least to some extent by their greater mapping specificity — the rate of increase of FP SNP numbers with read length in this experiment (Figs. 4 and 5) was not as pronounced as could be expected from what is theoretically possible (Fig. 8). Our original assumption was that longer reads map more specifically, thereby reducing the potential for mismapping. The expectation would then be that longer reads have lower rates of mismapping than shorter reads. Information about mismapping is readily available for this dataset due to our use of simulated reads which retain information about their origin in the read name. We analysed the rates of mismapping (i.e. the percentage of reads at SNP locations that contained the alternate allele and originated from a different chromosome or a different region on the same chromosome) for each call set and plotted these as a function of both read length and assembly (Fig. 10).

Interestingly, the relationship between read length and rates of mismapping appeared to depend on the reference sequence used. For the Allpaths-assembled reference and the controls, rates of mismapping appeared to decline with increasing read length (Fig. 10). For the Velvet-assembled reference sequences, this trend appeared to be reversed, and we currently have no explanation for this phenomenon.

The picture emerging from this is that there are probably two opposing forces involved here. On the one hand, there is the potential for longer reads to cause greater number of FP SNPs by introducing greater numbers of mismatches. On the other hand, we may have greater mapping specificity in longer reads, which means fewer reads get mismapped as read length increases, with an accompanying decrease in the likelihood of SNPs being called due to low alternate allele numbers. Within the current experiment, we did not simulate reads of the kind of lengths that are now being generated by e.g. the Pacific Biosciences and Oxford Nanopore technologies, and it would be highly interesting to explore in future experiments whether mapping reads of several kilobases in length genuinely improves mismapping.

### Genomic patterns of FP SNP locations

Regions containing FP SNPs were strongly enriched for transposable elements, reflecting the concentration of repeat elements in these regions and a large proportion of FP SNPs were located in the pericentromeric regions of the chromosomes, where such repetitive sequences are prevalent [26]. We conclude that misassembly or non-assembly of repeats or members of gene families in *de novo* genome assembly was the prime cause of FP SNPs in our study.

## Conclusions

Our experiment has highlighted and ranked multiple factors that have significant effects on the generation of FP SNPs during variant calling. First and foremost, the quality of the reference sequence is of paramount importance. Fragmentation, misassembly and non-assembly of regions within the reference sequence lead to read mapping targets being effectively unavailable, and the corresponding reads mapping to incorrect locations, leading to FP SNP accumulation. The second major determinant of FP SNP numbers in our experiment is the stringency of the read mapping, with relaxed mappings generally producing larger numbers of FP SNPs than strict mappings. However, these differences were found to be large only for the combination of Bowtie2, longer reads (300, 500, 1000 bp) and high quality reference sequence, and BWA with the poor quality reference sequences. This is an important finding, as both the mappers used here are supplied with relatively relaxed mismatch settings as defaults. We strongly discourage users from running read mappers on relaxed mismatch setting defaults to maximise the numbers of reads mapped. However, there is a caveat in that very strict mappings may lead to false negative SNPs, and more work is required to formulate an optimal approach to determining a mismatch rate that minimises both false positive and false negative SNPs.

The choice of mapper and variant caller also have significant effects upon FP SNP discovery, as does the use of MAPQ and depth filters for SNPs.

Read length was seen to play a comparatively minor role in FP SNP generation, with a complex relationship emerging between read length and FP SNP number. We conclude that the potential for greater mapping specificity in longer reads is at least partially offset by the increased numbers of mismatches they can contribute, which potentially translates into greater numbers of FP SNPs. Overall, we recommend that a good quality reference sequence is extremely important for mapping-based variant calling, along with stringent mappings and appropriate filtering of SNPs by at least MAPQ and coverage depth.

The above result highlights the importance of interactions among the factors in a SNP discovery pipeline. It is not sufficient just to specify individual parameter values in isolation, as these can be advantageous or disadvantageous depending upon the choice of the other factors.

## Additional file

Additional file 1 **Supplementary materials.** This supplementary pack file is comprised of the following ones: ANOVA_FullResults.xlsx – comprises the multifactorial ANOVA results; avgPctOfMismapping.xlsx – details the average percentages of reads containing the alternate allele and across the mappings; readMappingStats.xlsx – brings the alignment rates of reads, in the mappings, retrieved with the SAMtools flagstat command; snpNumbersStats.xlsx – details the SNP numbers computed in the experiment; SupplementalData.pdf – contains all the additional information and files mentioned in the manuscript. (ZIP 1362 kb)

## Abbreviations

ANOVA: Analysis of Variance
API: Application Programming Interface
bp: base pair(s)
CDS: Coding sequence
Chr: Chromosome
CPU: Central Processing Unit
FP: False positive
Java SE: Java Standard Edition
MAPQ: Mapping quality
Mbp: million base pairs
NGS: Next-generation sequencing
SAM: Sequence Alignment/Map file format
Sed: standard error of the difference
SNP: Single-nucleotide polymorphism
VCF: Variant Call Format
d.f.: degrees of freedom
s.s.: sum of squares
m.s.: mean square
v.r.: variance ratio
F prob.: F probability
perm prob.: permutation probability
Percentage SS: percentage of sum of squares
