# Supplementary material for: An investigation of causes of false positive single nucleotide polymorphisms using simulated reads from a small eukaryote genome
Source: BMC Bioinformatics. 2015 Nov 11;16:382. doi: 10.1186/s12859-015-0801-z (PMC4642669; doi:10.1186/s12859-015-0801-z)
Supplement: Supplementary file 1 — Supplementary materials. This supplementary pack file is comprised of the following ones: ANOVA_FullResults.xlsx – comprises the multifactorial ANOVA results; avgPctOfMismapping.xlsx – details the average percentages of reads containing the alternate allele and across the mappings; readMappingStats.xlsx – brings the alignment rates of reads, in the mappings, retrieved with the SAMtools flagstat command; snpNumbersStats.xlsx – details the SNP numbers computed in the experiment; SupplementalData.pdf – contains all the additional information and files mentioned in the manuscript. (ZIP 1362 kb) [file 12859_2015_801_MOESM1_ESM.zip › 12859_2015_801_MOESM1_ESM/SupplementalData.pdf]

An investigation of causes of false positive single nucleotide polymorphisms using simulated reads from a small eukaryote genome

Supplementary Data

Antonio Ribeiro Agnieszka Golicz Christine Anne Hackett Iain Milne Gordon Stephen David Marshall Andrew J Flavell Micha Bayer

July 07th, 2015

SD.1 Read simulation – additional information

SD.1.1 SimSeq configuration planning

The Allpaths-LG manual (revision of 27-Jan-13 2:47:00 PM), section “Supported library constructions”, and the work of Earl et al. (2011) were used as guidelines for setting up the parameter values for the read simulation stage. The calculations used for calculating the parameters are summarized in the following tables:

Table S1. Original genome sequence statistics

Chromosome	Number of base pairs (bp)
1	30,427,671
2	19,698,289
3	23,459,830
4	18,585,056
5	26,975,502
Total number of bases	119,146,348

Table S2. Coverage depth calculations

Coverage depth	Number of base pairs (bp)
50 x	5,957,317,400
100 x	11,914,634,800

Table S3. Number of reads per read length dataset

Read length dataset (bp)	Number of reads required (fragment library with 100x coverage depth)	Number of paired-end reads per FASTQ file (raw)	Number of paired-end reads per FASTQ file (rounded)
50	238,292,696	119,146,348	119,146,348
100	119,146,348	59,573,174	59,573,174
150	79,430,898.67	39,715,449.33	39,715,449
300	39,715,449.33	19,857,724.67	19,857,725
500	23,829,269.60	11,914,634.80	11,914,635
1,000	11,914,634.80	5,957,317.40	5,957,317
Read length dataset (bp)	Number of reads required (jumping library with 50x coverage depth)	Number of paired-end reads per FASTQ file (raw)	Number of paired-end reads per FASTQ file (rounded)
150	39,715,449.33	19,857,724.67	19,857,725

Table S4. Insert lengths and standard deviations per read length dataset calculations

Read length dataset (bp)	Fragment library short insert length (bp)	Standard deviation (bp)
50	90	9
100	180	18
150	270	27
300	540	54
500	900	90
1,000	1,800	180
Read length dataset (bp)	Jumping library short insert length (bp)	Standard deviation (bp)
150	3,000	300

SD.1.2 SimSeq configuration

SimSeq command usage as described at https://github.com/jstjohn/SimSeq:

```
Usage: java -jar -Xmx2048m SimSeq.jar [required options] [options]
            Last Updated: 4.12.2011

Usage example: java -jar –Xmx10g SimSeq.jar -1 50 -2 50 --
insert_size 90 --insert_stdev 9 --read_number 119146348 --
read_prefix 50bp_AT_SimSeq_1st_PE --reference TAIR10_All5Chrms.fasta
--inf_id --out 50bp_AT_SimSeq_1st_PE.sam

Usage example: java -jar –Xmx10g SimSeq.jar -1 150 -2 150 --
insert_size 3000 --insert_stdev 300 --mate_pair --mate_frag 500 --
mate_frag_stdev 50 –mate_pulldown_error_p 0.0 --read_number 19857725
--read_prefix 150bp_AT_SimSeq_1st_MP --reference
TAIR10_All5Chrms.fasta --inf_id --out 150bp_AT_SimSeq_1st_MP.sam


-1 <argument>                      Integer length of first read,
                                   accordingly to the aimed read
                                   length dataset in this case.

-2 <argument>                      Integer length of second read,
```

	accordingly to the aimed read length dataset in this case.
--insert_size <argument>	Mean library insert size for either mate-paired or paired-end reads, accordingly to the aimed read length dataset in this case.
--insert_stdev <argument>	Mean library insert standard deviation for either mate-paired or paired-end reads, accordingly to the aimed read length dataset in this case.
--read_number <argument>	Integer number of reads to be sampled, in order to provide 100-fold coverage depth, accordingly to the aimed read length dataset in this case.
--read_prefix <argument>	Prefix for the simulated reads (e.g. 50bp_AT_SimSeq_1st_PE).
--reference <argument>	Reference genome sequence file in uncompressed FASTA format. (REQUIRED)
--inf_id	Flag to output location information in the read identifier.
--out <argument>	Filename for output SAM file. (REQUIRED)

Jumping library dataset specific options:

--mate_pair	Flag to perform mate-pair rather than paired-end run.
--mate_frag <argument>	Loop fragmentation size set up with the default value of 500.
--mate_frag_stdev <argument>	Loop fragmentation standard deviation set up with the default value of 50.
--mate_pulldown_error_p <argument>	Probability that a read does not include the biotin marker set up as 0.0 in this case.
--read_number <argument>	Integer number of reads to be sampled, in order to provide 50-fold coverage depth for the aimed 150 bp read length dataset in

	this case.

In order to emulate a Phred quality score of 40 (considering Phred+33 scale) evenly for all the bases across the read length datasets, the base quality scores in the intermediate SAM files produced within the SimSeq pipeline had the "~" character substituted with an "I", using the Unix sed command. After these substitutions, the SimSeq usage example, available at https://github.com/jstjohn/SimSeq, served as a guideline for the remaining steps of the pipeline for the production of the simulated reads, in FASTQ format, corresponding to each read length dataset. The steps performed were as follows:

- Creation of the ".size" file for the SAM to BAM format conversion:

```
Usage: faSize [command flags] file(s).fa

Usage example:  ./SimSeq-master/cUtils/faSize –detailed -tab
<in.fa> > <out.size>
```

-detailed	Flag to output name and size of each record.
-tab	Flag to output statistics in a tab separated format.
<in.fa>	Original sequences input file in FASTA format.
<out.size>	Desired output file labelled with .size extension in this case.

- SAM to BAM format conversion:

```
Usage:  samtools view [options] <in.bam>|<in.sam> [region1
[…]]

Usage example: samtools view -bS –T <in.reference> -t
<in.size> -o <out.bam> <in.sam>
```

-b	Flag to output BAM.
-S	Flag to specify that input is SAM.
-T <in.reference>	Reference sequence file.
-t <in.size>	List of reference names and lengths (.size file in this case).
-o <out.bam>	Output .bam filename.
<in.sam>	Input .sam file.

- BAM file sorting:

```
Usage:  samtools sort [options] <in.bam> <out.prefix>

Usage example: samtools sort <in.bam> <out.sorted>
```

`<in.bam>`	Input .bam file.
`<out.sorted>`	Output sorted .bam filename.

- Sorted BAM file indexing:

```
Usage:  samtools index <in.bam> [out.index]

Usage example: samtools index <in.sorted.bam>
```

`<in.sorted.bam>`	Input sorted .bam file.

- SAM to FASTQ conversion:

```
Usage:  SamToFastq [options]

Usage example: java -jar -Xmx10g ./picard-tools-
1.119/SamToFastq.jar INPUT=File FASTQ=File
SECOND_END_FASTQ=File INCLUDE_NON_PF_READS=true
VALIDATION_STRINGENCY=SILENT
```

INPUT=File	Input sorted .bam file to extract reads from. (REQUIRED)
FASTQ=File	Output FASTQ file (single-end FASTQ or, if paired, first end of the pair FASTQ). (REQUIRED)
SECOND_END_FASTQ=File	Output FASTQ file (if paired, second end of the pair FASTQ).
INCLUDE_NON_PF_READS=true	Include non-PF reads from the SAM file into the output FASTQ files. PF means 'passes filtering'. Reads whose 'not passing quality controls' flag is set are non-PF reads.
VALIDATION_STRINGENCY=SILENT	Opted validation stringency for all SAM files read by this program.

SD.2 *De novo* assembly – additional information

SD.2.1 Velvet configuration

Velvet command usage was based on the software package embedded manual version 1.1 and a specific recommendation about mate-paired sequences usage described at http://thegenomefactory.blogspot.co.uk/2012/09/using-velvet-with-mate-pair-sequences.html. Due to this, mate-paired reads were initially reverse-complemented using EMBOSS revseq tool (http://emboss.sourceforge.net/apps/release/6.3/emboss/apps/revseq.html) version EMBOSS:6.6.0.0:

- revseq step:

```
Usage: revseq [-sequence] <argument> -sformat1 <argument> [-outseq]
<argument> -osformat2 <argument> -[no]tag

Usage example: revseq -sequence 150bp_AT_SimSeq_1st_MP_1.fastq -
sformat1 fastq-sanger -outseq rc_150bp_AT_SimSeq_1st_MP_1.fastq -
osformat2 fastq-sanger -notag

Usage example: revseq -sequence 150bp_AT_SimSeq_1st_MP_2.fastq -
sformat1 fastq-sanger -outseq rc_150bp_AT_SimSeq_1st_MP_2.fastq -
osformat2 fastq-sanger -notag
```

```
[-sequence] <argument>          (Gapped) nucleotide sequence(s)
                                filename and optional format, or
                                reference (input USA). (REQUIRED)

-sformat1 <argument>            Input sequence format. In this
                                case, fastq-sanger.

[-outseq] <argument>            [<sequence>.<format>] Sequence
                                set(s) filename and optional
                                format (output USA). (REQUIRED)

-osformat2 <argument>           Output sequence format. In this
                                case, fastq-sanger.

-[no]tag                        [Y] Set this to be false if you
                                do not wish to add 'Reversed:' to
                                the sequence description.
```

- velveth step:

```
Usage: ./velveth directory hash_length {[-file_format][-read_type][-
separate|-interleaved] filename1 [filename2 ...]} {...} [options]

Usage example: velveth . 96 -shortPaired -separate -fastq
150bp_AT_SimSeq_1st_PE_1.fastq 150bp_AT_SimSeq_1st_PE_2.fastq -
shortPaired2 -separate -fastq rc_150bp_AT_SimSeq_1st_MP_1.fastq
rc_150bp_AT_SimSeq_1st_MP_2.fastq
```

directory
Directory name for output files.

hash_length
Odd integer (if even, it will be decremented) <= 99 (if above, will be reduced).
Note: although the parameter was input as 96, in order to make it as most comparable as possible with the intrinsic hash length used by the Allpaths-LG assembler in this study case, the hash length value was automatically decremented to 95 by the velveth package.

[-file_format]
File format option. In this case, –fastq.

[-read_type]
Read type option. In this case, -shortPaired.

[-separate|-interleaved]
File layout options for paired reads (only for FASTA and FASTQ formats). In this case, -separate, meaning “read 2 separate files for paired reads”.

filename1
Path to sequence file in this case.

[filename2 ...]
Path to second sequence file in this case.

- velvetg step:

Usage: ./velvetg directory [options]

Usage example: velvetg . -read_trkg yes -amos_file yes -exp_cov auto -cov_cutoff auto -shortMatePaired2 yes

directory
Working directory name.

-read_trkg <yes|no>
Tracking of short read positions in assembly. In this case, yes.

-amos_file <yes|no>
Export assembly to AMOS file. In this case, yes.

-exp_cov <floating point|auto>
Expected coverage of unique regions or allow the system to infer it. In this case, auto.

```
-cov_cutoff <floating-point|auto>  Removal of low coverage nodes
                                   AFTER tour bus or allow the
                                   system to infer it. In this case,
                                   auto.

-shortMatePaired* <yes|no>         For mate-pair libraries, indicate
                                   that the library might be
                                   contaminated with paired-end
                                   reads. In this case,
                                   -shortMatePaired2 yes.
                                   Note: Parameter included here
                                   just for the sake of consistency
                                   with the (mentioned above)
                                   recommendation about usage of
                                   "mate-paired sequences" with
                                   Velvet.
```

SD.2.2 Allpaths-LG configuration

Allpaths-LG command usage was based on the software package embedded manual revision of 27-Jan-13 2:47:00 PM:

- Input files examples obtained after performing the steps described in the manual's section "Preparing data for ALLPATHS":

in_groups.csv

```
group_name,library_name,file_name
1,150bp_AT_SimSeq_1st_PE,
/mnt/scratch/ar41690/1st_AT_SimSeq/150bp_AT_SimSeq_1st_PE_*.fastq
2,150bp_AT_SimSeq_1st_MP,
/mnt/scratch/ar41690/1st_AT_SimSeq/150bp_AT_SimSeq_1st_MP_*.fastq
```

in_libs.csv

```
library_name,project_name,organism_name,type,paired,frag_size,frag_stddev,i
nsert_size,insert_stddev,read_orientation,genomic_start,genomic_end
150bp_AT_SimSeq_1st_PE,AT_AllpathsLG_1stAssy,A.thaliana,fragment,1,270,27,,
,inward,,
150bp_AT_SimSeq_1st_MP,AT_AllpathsLG_1stAssy,A.thaliana,jumping,1,,,3000,30
0,outward,,
```

ploidy

```
1
```

- Perl script "PrepareAllPathsInputs.pl" run:

```
Usage: PrepareAllPathsInputs.pl
DATA_DIR=<full_path to REFERENCE DIR>/mydata
PICARD_TOOLS_DIR=/opt/picard/bin
```

```
Usage example: PrepareAllPathsInputs.pl
DATA_DIR=/mnt/scratch/ar41690/
1st_AT_SimSeq/AT_AllpathsLG_1stAssy/REFERENCE/DATA/
```

DATA_DIR=<full_path to REFERENCE DIR>/mydata	Target data directory.

- "RunAllPathsLG" pipeline run:

```
Usage: RunAllPathsLG arg1=value1 arg2=value2 ...

Usage example: RunAllPathsLG
PRE=/mnt/scratch/ar41690/1st_AT_SimSeq/AT_AllpathsLG_1stAssy/
DATA_SUBDIR=DATA RUN=RUN REFERENCE_NAME=REFERENCE TARGETS=standard
```

PRE=<full_path to PRE DIR>	The root directory in which the ALLPATHS pipeline directory will be created.
DATA_SUBDIR=<DATA directory name>	The DATA (project) directory name.
RUN=<RUN directory name>	The RUN (assembly pre-processing) directory name.
REFERENCE_NAME=<REFERENCE directory name>	The REFERENCE (organism) directory name.
TARGETS=<value>	Determines the operations performed by the pipeline. In this case, standard.

Table S5. QUAST results for each assembly replicate

Assembly detail	First Velvet assembly	Second Velvet assembly	First Allpaths-LG assembly	Second Allpaths-LG assembly
# contigs (>= 0 bp)	4,251	4,252	607	586
# contigs (>= 1,000 bp)	403	401	607	586
Total length (>= 0 bp)	119,084,054	119,053,539	115,964,030	115,898,343
Total length (>= 1,000 bp)	117,876,165	117,840,546	115,964,030	115,898,343
# contigs	842	845	607	586
Largest contig	8,578,385	6,688,511	3,993,636	3,183,886
Total length	118,169,135	118,138,303	115,964,030	115,898,343
Reference length	119,146,348	119,146,348	119,146,348	119,146,348
GC (%)	36.01	36.01	35.99	35.99
Reference GC (%)	36.03	36.03	36.03	36.03
N50	2,924,849	2,808,495	901,890	844,369
NG50	2,855,571	2,808,495	883,049	838,397
N75	1,400,402	1,400,345	344,027	349,933
NG75	1,400,402	1,400,345	324,538	321,283
L50	11	13	37	37
LG50	12	13	39	39
L75	26	28	88	88
LG75	26	28	95	95
# misassemblies	132	145	183	191
# misassembled contigs	54	57	115	110
Misassembled contigs length	71,484,825	60,248,624	50,403,708	56,506,149
# local misassemblies	726	721	835	828
# unaligned contigs	0 + 46 part	0 + 35 part	0 + 1 part	0 + 0 part
Unaligned length	147,154	107,527	505	0
Genome fraction (%)	98.324	98.332	96.479	96.416
Duplication ratio	1.008	1.008	1.009	1.009
# N's per 100 kbp	574.51	537.53	665.70	654.05
# mismatches per 100 kbp	5.16	5.37	5.89	6.35
# indels per 100 kbp	0.42	0.42	1.17	1.23
# genes	28,186 + 113 part	28,185 + 111 part	27,967 + 267 part	27,970 + 263 part
Largest alignment	7,477,918	5,889,670	3,000,193	3,177,551
NA50	1,934,540	2,422,643	693,004	615,806
NGA50	1,934,540	2,422,643	663,650	609,210
NA75	1,025,291	988,328	268,585	255,409
NGA75	1,005,107	980,170	236,584	236,667
LA50	16	15	45	48
LGA50	16	15	48	50
LA75	36	34	111	121
LGA75	37	35	121	131

SD.3 Read mapping – additional information

To prevent technical problems in the downstream analysis, all read names were changed so the read identifiers would end in the suffixes "_R1" and "_R2" instead of the original "/1" and "/2" assigned by the read simulator. This was done using the Unix "sed" tool. Also, the Unix "tr" command and custom Java code were used to check for any occurrences of IUPAC ambiguity codes (Cornish-Bowden, 1985) in the read datasets and in the *de novo* and control assemblies, replacing them with "N" characters as necessary. Mappings were then performed using Bowtie2 and BWA-SW set up with the following parameters to map with the two mismatch stringency rates (2% and 14%) evaluated in the study:

SD.3.1 BWA-SW configuration

The mapping stringency in BWA-SW is controlled by means of the minimum score threshold (-T parameter, see http://bio-bwa.sourceforge.net/bwa.shtml#3). Reads with a mapping score of less than -T will not be mapped. Based on observations from BWA-SW's SAM output we concluded that the maximum score is equal to the length of the read, and, for each mismatch, 5 units are deducted. The value for -T needs to be calculated separately for each read length.

Example with a 100 bp read:

max. score = 100
-T score threshold = 30
subtract threshold from max. score: 100 - 30 = 70
divide difference by the penalty awarded for each mismatch: 70/5 = 14 mismatches

However, the actual computation of the alignment score does seem to vary slightly with read length. To establish actual values for -T, we made input data sets with 3 reads each for each read length, and corresponding reference sequences (data not shown). In each case, read 1 matched the reference perfectly, read 2 had the allowed number of mismatches based on a 2% mismatch rate, and read 3 had one more mismatch than allowed and should therefore be rejected. The actual cut-offs, based on the alignment scores observed in the SAM output, were slightly different from the theoretical values, as shown in Table 6. The values for -T actually used for the experiment were those shown in the last column of each table.

Table S6. Values for BWA-SW -T parameter (last column) – 2% mismatches

Read length (bp)	Number of mismatches allowed	max. score value possible	max. -T value allowed (THEORETICAL)	max. -T value allowed (ACTUAL)
50	1	50	45	45
100	2	100	90	90
150	3	150	135	135
300	6	300	270	275
500	10	500	450	460
1,000	20	1,000	900	920

Table S7. Values for BWA-SW -T parameter (last column) – 14% mismatches

Read length (bp)	Number of mismatches allowed	max. score value possible	max. -T value allowed (THEORETICAL)	max. -T value allowed (ACTUAL)
50	7	50	15	23
100	14	100	30	45
150	21	150	45	65
300	42	300	90	132
500	70	500	150	220
1,000	140	1,000	300	440

- BWA-SW parameters used:

```
Usage: bwa bwasw [options] <target.prefix> <query.fa> [query2.fa]

Usage example: bwa bwasw -T <int> -t <int> <in.db.fasta> <in.fq>
<mate.fq> > <out.sam>

-T <int>              Score threshold divided by a.

-t <int>              Number of threads.

<in.db.fasta>         Path to the file containing the
                      reference assembly in FASTA
                      format.

<in.fq>               Path to the file containing the
                      first paired-end reads dataset in
                      FASTQ format.

<mate.fq>             Path to the file containing the
                      second paired-end reads dataset
                      in FASTQ format.

<out.sam>             Output .sam filename.
```

For BWA-SW SAM files, read group tags and headers were added with a custom script as this was required by the GATK downstream analysis.

SD.3.2 Bowtie2 configuration

The mismatch rate in Bowtie2 can be controlled with the --score-min parameter. From the Bowtie2 manual (http://bowtie-bio.sourceforge.net/bowtie2/manual.shtml#bowtie2-options-score-min):

"This is a function of read length. For instance, specifying L,0,-0.6 sets the minimum-score function f to f(x) = 0 + -0.6 * x, where x is the read length."

The first parameter ("L" in the example above) specifies a linear relationship between read length and the number of mismatches. The second parameter (0 in the example above) is the y intercept, and, the third parameter, the coefficient for the slope of the regression. To

calculate the coefficient for the formula, we divide the intended maximum mismatch score (obtained by multiplying the number of mismatches by the default penalty -6) by the read length, like in this example which assumes a read length of 100 bp and a maximum number of two mismatches per read:

Coeff = (-6*2)/100 = -0.12

Thus, the score-min formula for Bowtie2 for the strict 2% mismatch rate used here was "L,0,-0.12", whereas the relaxed mismatch rate of 14% used a score-min of "L,0, -0.84" (=(-6*14)/100).

- Bowtie2 parameters used:

```
Usage: bowtie2 [options]* -x <bt2-idx> {-1 <m1> -2 <m2> | -U <r>} [-S <sam>]

Usage example: bowtie2 -x <bt2-idx> -1 <m1> -2 <m2> -S <sam> --phred33 --score-min <func> -p <int> --rg-id <text> --rg <text>
```

-x <bt2-idx>	Index filename prefix (minus trailing .X.bt2).
-1 <m1>	Files with #1 mates, paired with files in <m2>.
-2 <m2>	Files with #2 mates, paired with files in <m1>.
-S <out.sam>	File for SAM output.
--phred33	Qualities are Phred+33.
--score-min <func>	Min. acceptable alignment score w/r/t read length.
-p <int>	Number of alignment threads to launch.
--rg-id <text>	Set read group id, reflected in @RG line and RG:Z: opt field.
--rg <text>	Add <text> ("lab:value") to @RG line of SAM header.

All BAM files were checked for multimapped reads and, where necessary, these were filtered out via a custom script using SAMtools 0.1.18 (http://samtools.sourceforge.net/) and Picard Tools 1.119 (http://broadinstitute.github.io/picard). The latter was also used to, where necessary, filter out soft-clipped reads at the end of contigs. The range of percentages of discarded reads in the BAM files which had some sort of filtering applied was of 0.01% to 0.13%.

Additional information about the mappers and their parameters can be found at http://bio-bwa.sourceforge.net/bwa.shtml and http://bowtie-bio.sourceforge.net/bowtie2/manual.shtml.

SD.4 SNP calling – additional information

SD.4.1 FreeBayes configuration

The FreeBayes command line parameters used are described below:

```
Usage: freebayes/0.9.18/bin/freebayes [OPTION] ... [BAM FILE] ...

Usage example: /mnt/apps/freebayes/0.9.18/bin/freebayes -b <in.bam>
-f <in.reference> -v <out.vcf> -q <Q> -m <Q> -Z
```

-b <in.bam>	Add FILE to the set of BAM files to be analyzed.
-f <in.reference>	Use FILE as the reference sequence for analysis.
-v <out.vcf>	Output VCF-format results to FILE.
-q <Q>	Minimum base quality input filter. Exclude alleles from analysis if their supporting base quality is less than Q. In the study, this value has been always set as 10.
-m <Q>	Minimum mapping quality input filter. Exclude alignments from analysis if they have a mapping quality less than Q. In the study, this value has been set as 0 or 20, depending on the combination of factors being evaluated.
-Z	Include the reference allele in the analysis.

Additional information can be found at https://github.com/ekg/freebayes and in the FreeBayes embedded –help option.

SD.4.2 GATK configuration

The parameters used are described below, for each of the components integrated in our pipeline script:

- Picard Tool's MarkDuplicates step:

```
Usage: MarkDuplicates [options]

Usage example: java -jar <pathToPicard>/MarkDuplicates.jar
INPUT=<File> OUTPUT=<File> METRICS_FILE=<File> AS=<Boolean>
MAX_FILE_HANDLES_FOR_READ_ENDS_MAP=<Integer>
```

INPUT=<File>	One or more input SAM or BAM files to analyze.
OUTPUT=<File>	The output file to write marked records to. (REQUIRED)
METRICS_FILE=<File>	File to write duplication metrics to. (REQUIRED)
AS=<Boolean>	If true, assume that the input file is coordinate sorted even if the header says otherwise. In the study, this value has been always set as true.
MAX_FILE_HANDLES_FOR_READ_ENDS_MAP=<Integer>	Maximum number of file handles to keep open when spilling read ends to disk. In the study, this value has been always set as 1000.
READ_NAME_REGEX=<String>	Regular expression that can be used to parse read names in the incoming SAM file. In the study, for BWA-SW related input BAM files, this value has been always set as null to suppress the functionality of the parameter.
VALIDATION_STRINGENCY=<String>	Validation stringency for all SAM files read by this program. In the study, for BWA-SW related input BAM files, this value has been always set as LENIENT to allow the

processing to clear the MarkDuplicates stage.

- SAMTools BAM file indexing step:

```
Usage: samtools index <in.bam> [out.index]
```

```
Usage example: samtools index <in.deduped.bam>
```

<in.deduped.bam>	The output file produced by the MarkDuplicates step.

- GATK's realignment step 1 – target interval list generation:

Usage example:

```
java -jar <pathToGATK>/GenomeAnalysisTK.jar -T
RealignerTargetCreator -R <reference_sequence> -I <input_file> -o
<out> -nt <num_threads>
```

-R <reference_sequence>	Reference sequence file.
-I <input_file>	Input file containing sequence data. In this case, the output BAM file produced by the MarkDuplicates step.
-o <out>	An output file created by the walker. In this case, the target_intervals.list file to be created in this step.
-nt <num_threads>	Number of data threads to allocate to this analysis.

- GATK realignment step 2 – Indel realignment:

Usage example:

```
java -jar <pathToGATK>/GenomeAnalysisTK.jar -T IndelRealigner -R
<reference_sequence> -I <input_file> -targetIntervals
<targetIntervals> -o $uniquePrefix.realigned.bam
```

-R <reference_sequence>	Reference sequence file.
-I <input_file>	Input file containing sequence data. In this

	case, the output BAM file produced by the MarkDuplicates step.
targetIntervals <targetIntervals>	Intervals file output from RealignerTargetCreator step.
-o <out>	Output BAM file. In this case, the realigned BAM file to be created in this step.

- GATK HaplotypeCaller step:

Usage example:

```
java -jar <pathToGATK>/GenomeAnalysisTK.jar -T HaplotypeCaller -R
<reference_sequence> -I <input_file> -o <out> -nct
<num_cpu_threads_per_data_thread> -mmq <min_mapping_quality_score>
```

-R <reference_sequence>	Reference sequence file.
-I <input_file>	Input file containing sequence data. In this case, the realigned BAM file produced by the IndelRealigner step.
-o <out>	File to which variants should be written.
-nct <num_cpu_threads_per_data_thread>	Number of CPU threads to allocate per data thread.
-mmq <min_mapping_quality_score>	Minimum read mapping quality required to consider a read for analysis with the HaplotypeCaller. In the study, this value has been set as 0 or 20, depending on the combination of factors being evaluated.

Additional information can be found at https://github.com/ekg/freebayes, https://www.broadinstitute.org/gatk/, http://broadinstitute.github.io/picard/index.html, and in the embedded `-h` option of each tool.

SD.5 An example of mismapping identified by the read origin information available from the read simulator

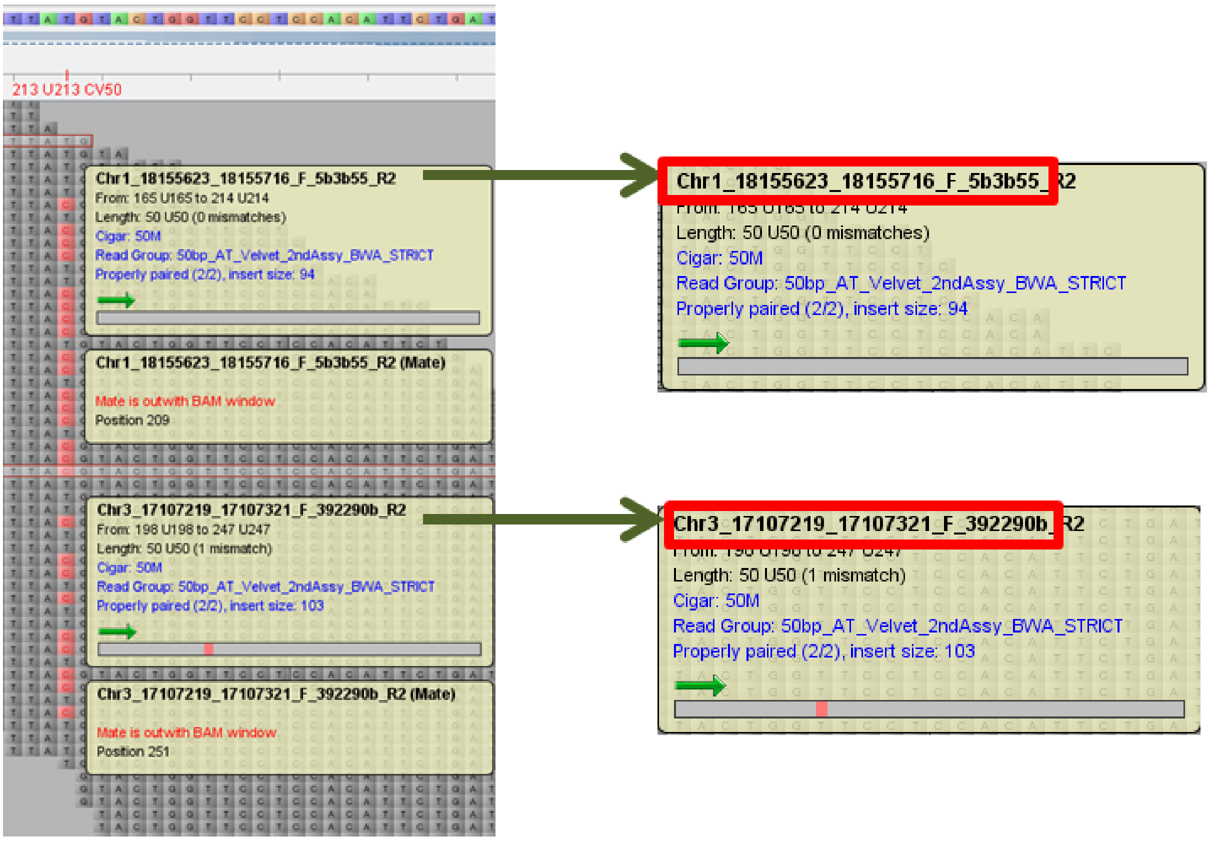

Fig. 1. Screenshots, from the Tablet assembly viewer (Milne, Bayer, *et al.*, 2010; Milne, Stephen, *et al.*, 2013), showing an example of a false positive SNP caused by read mismapping. The detail is showing the SimSeq read labels. The read with the alternate allele belongs to a different chromosome than those with the reference allele.

SD.6 Read mismapping quantification workflow

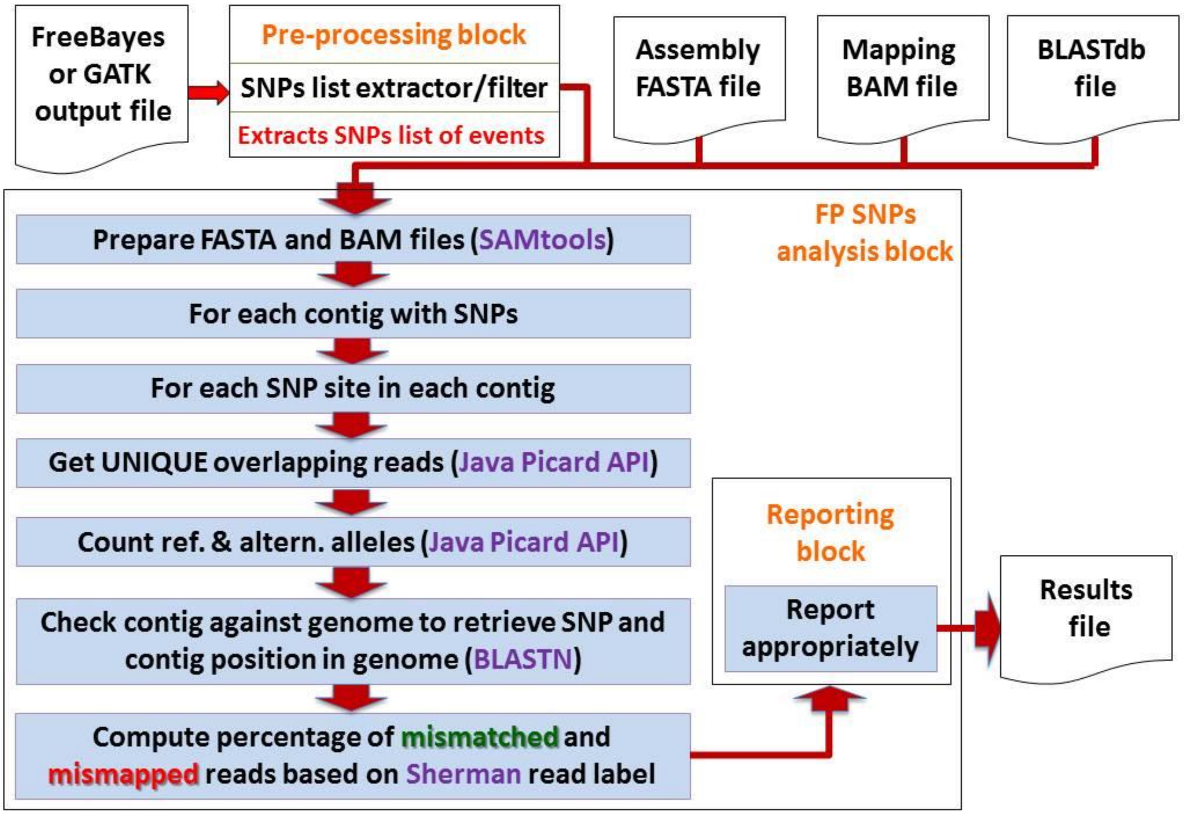

Fig. 2. Workflow of mismapped read quantification. The program scans for each SNP within a contig and extracts the reads overlapping (covering) each SNP site. It then counts the number of reads containing the same allele as the reference sequence, as well as the number of reads containing alternate alleles. It also checks for the original position and allele in the original genome that corresponds to the SNP site in the contig (with the BLASTN parameter -max_hsps_per_subject set to 1). Finally, it computes the percentage of mismapped reads containing the allele alternate to the corresponding allele in the genome, writing the output to a results file. We observed cases (approximately 63%, in average, across the assembly replicate runs) where there was disagreement between the allele in the assembled contig and the corresponding position in the original genome sequence (based on the BLASTN analysis), and, in such cases, we assumed that this was due to a single base misassembly in the contig. In these situations, the reads containing the allele observed in the contig were counted as mismatched, as they contained the allele that differed from the original genome sequence.

SD.7 BLAST database build for the SNP annotation stage

The following steps were performed to build the database the SNP manifests were queried against:

(1) The files ftp://ftp.arabidopsis.org/home/tair/Sequences/blast_datasets/TAIR10_blastsets/TAIR10_seq_20110103_representative_gene_model_updated and ftp://ftp.arabidopsis.org/home/tair/Sequences/blast_datasets/TAIR10_blastsets/TAIR10_intergenic_20101028 were downloaded and combined as a single FASTA file containing both coding and intergenic sequences.
(2) This file was used to build the BLAST database for querying the SNP manifests, using the *makeblastdb* command.

SD.7.1 SNP manifest extraction

The following steps were executed to extract the SNP manifests:

(1) All SNP entries passing the depth filter that belonged to a given assembly replicate (and considering any combination of factors and mapping/SNP calling scenario), were combined to form a single VCF file.
(2) The UNIX commands "cat" and "sort" were used to sort this VCF file, so the *vcfuniq* executable (https://github.com/ekg/vcflib) could be run on it.
(3) The resulting non-redundant VCF file was then processed with custom Java code, so the corresponding SNP manifests could be extracted from the assembly file.

The same procedure was performed for the 'control' dataset.

SD.8 SNP numbers detailed results

The SNP numbers computed in the experiment are detailed in the supplementary data file “snpNumbersStats.xlsx”.

SD.9 Overall alignment rates of reads

The SAMTools flagstat command was used to retrieve the alignment rates of reads in the mappings. Results are shown in the supplementary data file “readMappingStats.xlsx”.

SD.10 Read mismapping detailed results

The supplementary data file “avgPctOfMismapping.xlsx” details the percentages of reads containing the alternate allele and mismapped, averaged across the mappings.

SD.11 SNP annotation detailed results

After performing the BLAST of the SNP manifests from each assembly replicate and the control reference sequence, the Unix "awk" tool was applied to remove redundant lines of each corresponding BLAST result file, based on the "qseqid" field. Using Microsoft Excel version 14.0.7149.5000, a list of annotation terms was searched for and quantified from the "salltitles" field. A similar approach was used to categorise and quantify the same terms from the available *A. thaliana* annotation. These were then compared, and the following table and charts contain the results:

Table S8. *Arabidopsis thaliana* annotation general composition versus unique SNP manifests

	Number of occurrences retrieved by the used annotation approach					
Categories defined for SNP characterization	BLAST database	Allpaths-LG first run	Allpaths-LG second run	Velvet first run	Velvet second run	Controls (compiled)
family	10,530	5,030	5,053	2,427	2,523	1,317
intergenic	31,342	57,798	57,516	49,018	48,478	26,494
other CDS	13,115	5,347	5,137	2,368	2,269	993
pseudogene	876	1,345	1,044	523	538	346
repeat	1,410	407	611	303	345	131
reverse transcriptase	24	29	20	18	18	15
specific transposon / retrotransposon	20	-	-	-	-	-
transposable element gene	3,900	33,751	35,064	22,795	22,831	9,578
transposase	14	41	61	44	33	-
unknown protein	3,713	1,314	1,306	1,173	1,242	890
Totals	64,944	105,062	105,812	78,669	78,277	39,764

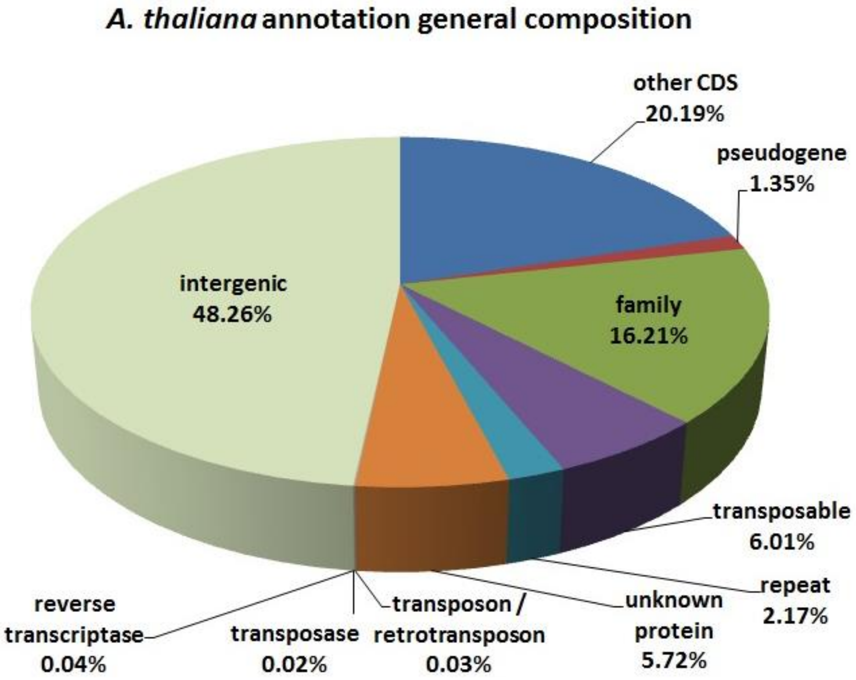

Fig. 3. General composition of the *Arabidopsis thaliana* annotation, as already shown in the main paper's Results section.

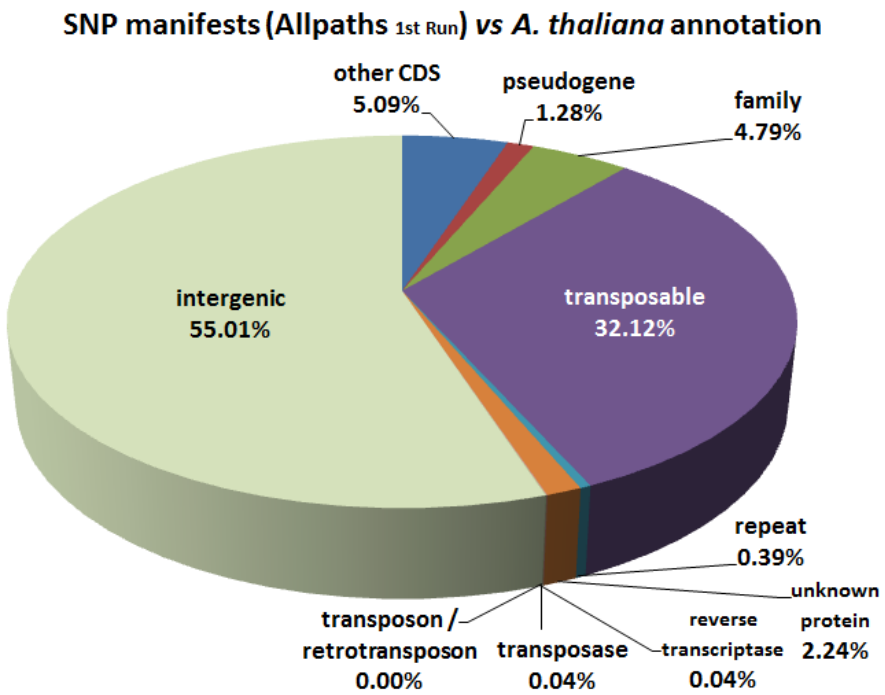

Fig. 4. BLAST-based annotation results for the SNP manifests from the Allpaths-LG first replicate of the experiment, as already shown in the main paper's Results section.

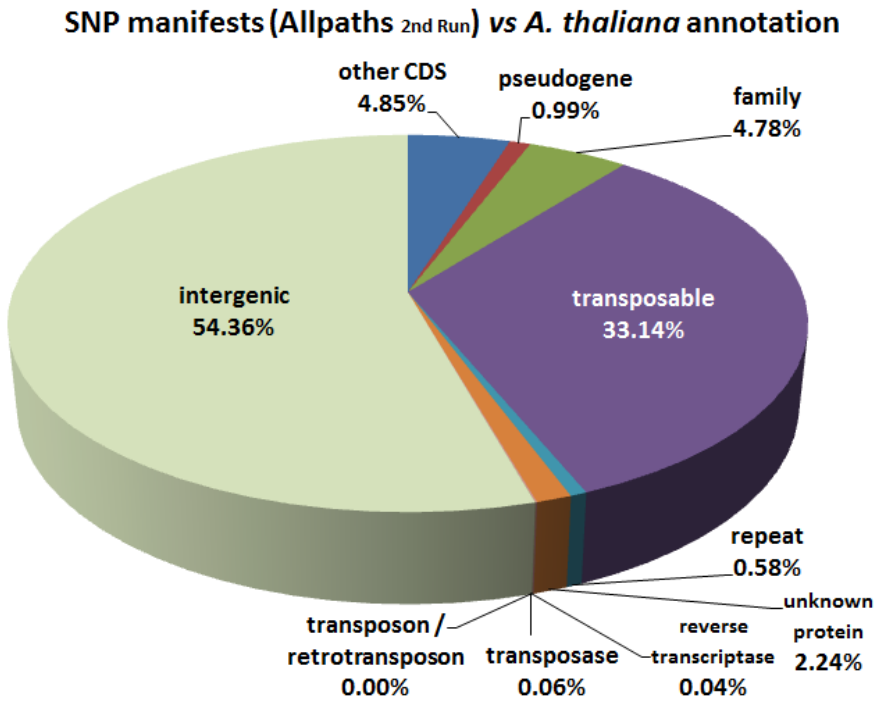

Fig. 5. BLAST-based annotation results for the SNP manifests from the Allpaths-LG second replicate of the experiment.

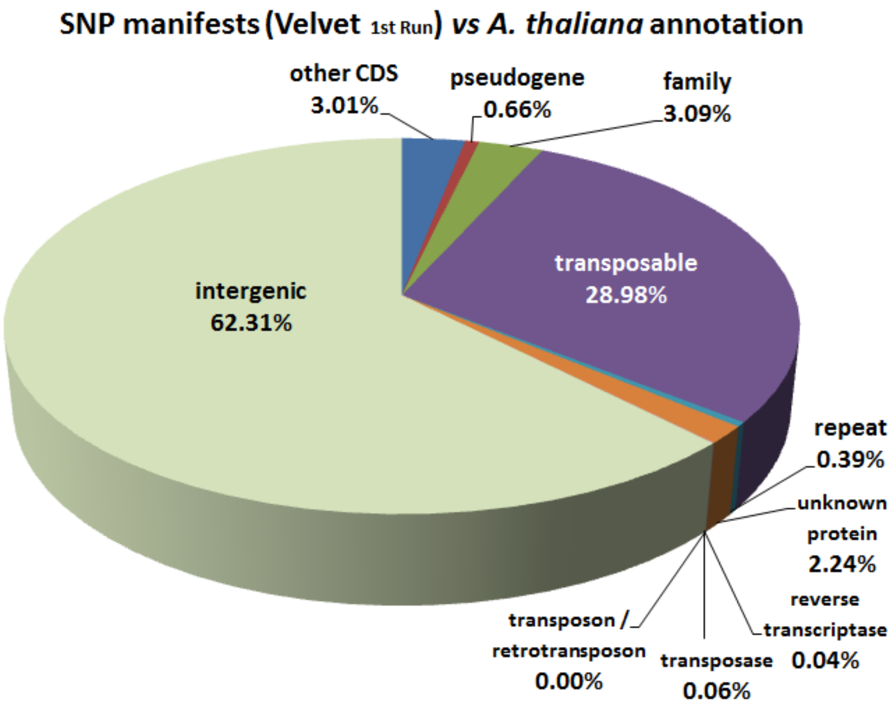

Fig. 6. BLAST-based annotation results for the SNP manifests from the Velvet first replicate of the experiment, as already shown in the main paper's Results section.

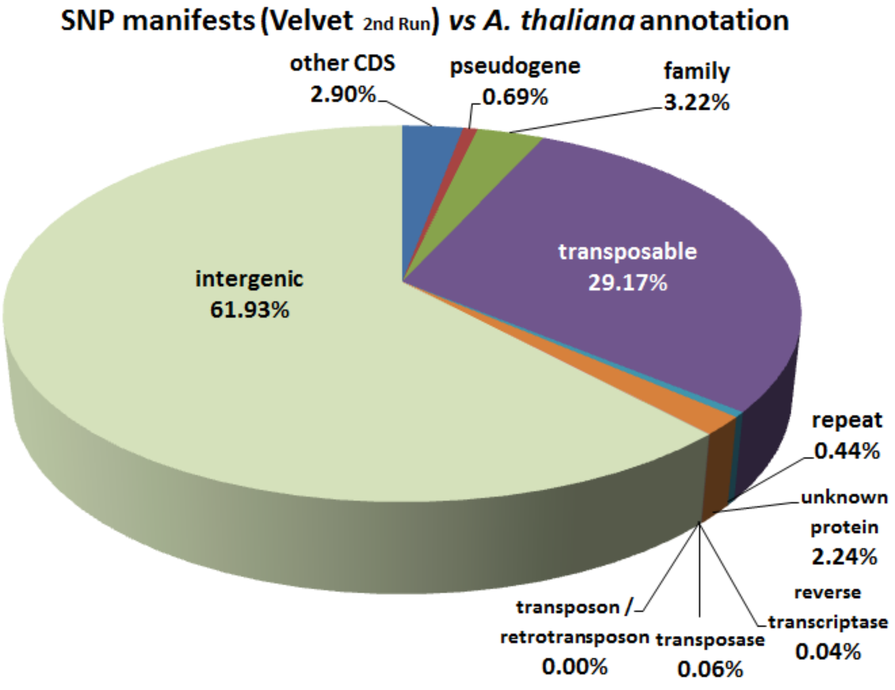

Fig. 7. BLAST-based annotation results for the SNP manifests from the Velvet second replicate of the experiment.

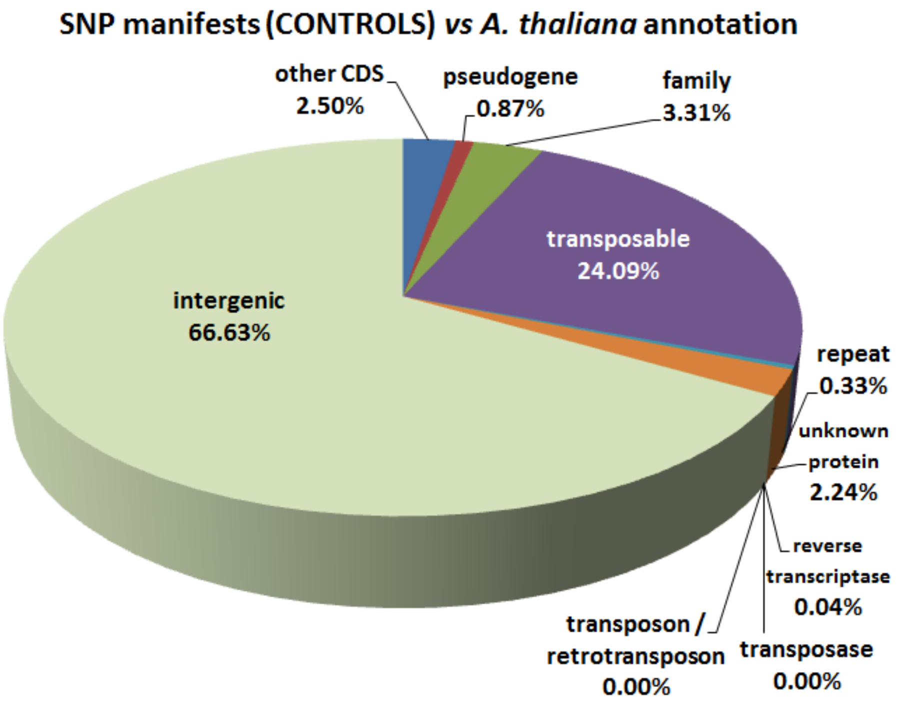

Fig. 8. BLAST-based annotation results for the SNP manifests from the two controls (compiled) of the experiment, as already shown in the main paper's Results section.

SD.12 SNP locations by chromosomes

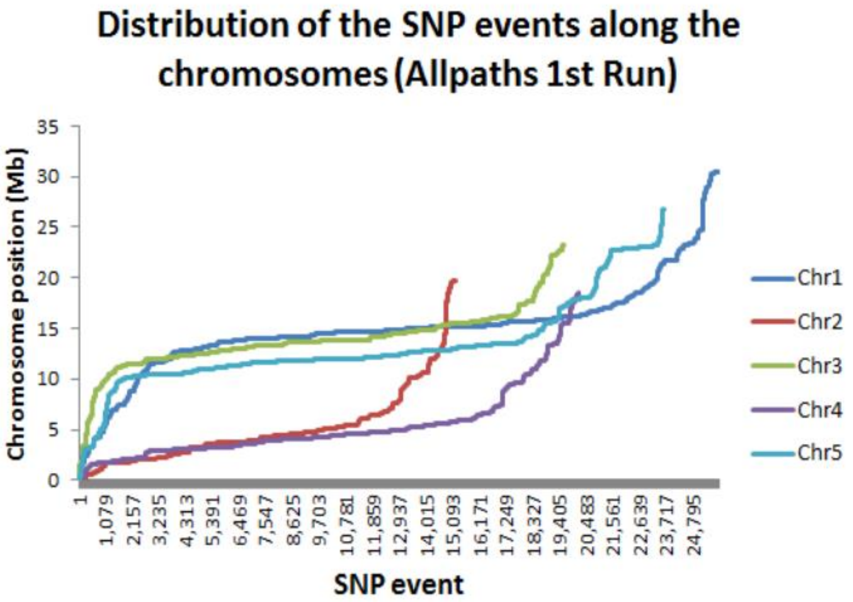

Fig. 9. Plot of SNP locations by chromosome, from the Allpaths-LG first replicate of the experiment. SNP events on the y axis are ordered by their position on the chromosome.

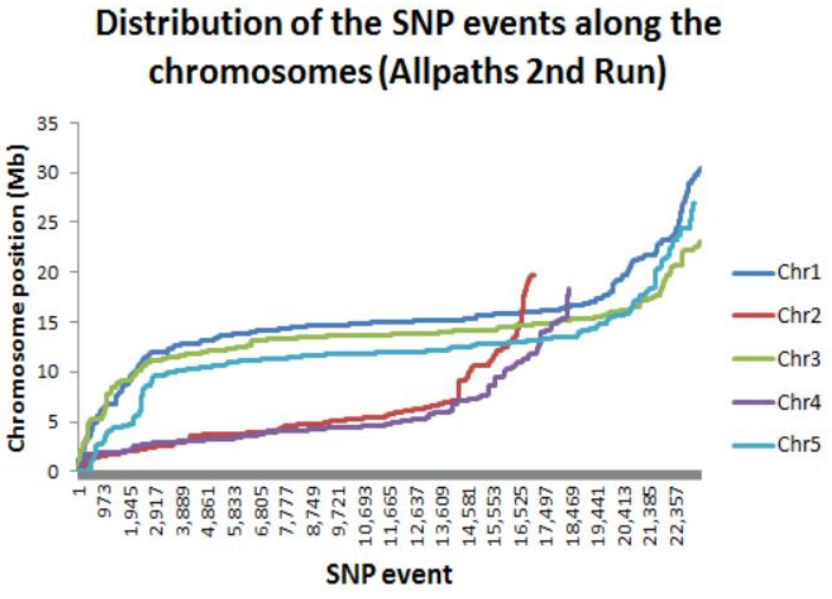

Fig. 10. Plot of SNP locations by chromosome, from the Allpaths-LG second replicate of the experiment. SNP events on the y axis are ordered by their position on the chromosome.

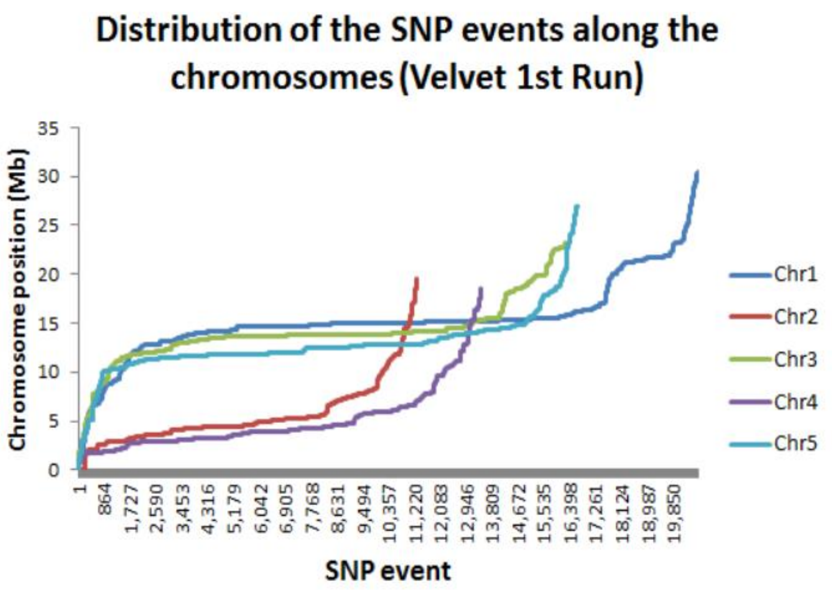

Fig. 11. Plot of SNP locations by chromosome, from the Velvet first replicate of the experiment (already shown in the main paper's Results section, but illustrated with different legend). SNP events on the y axis are ordered by their position on the chromosome.

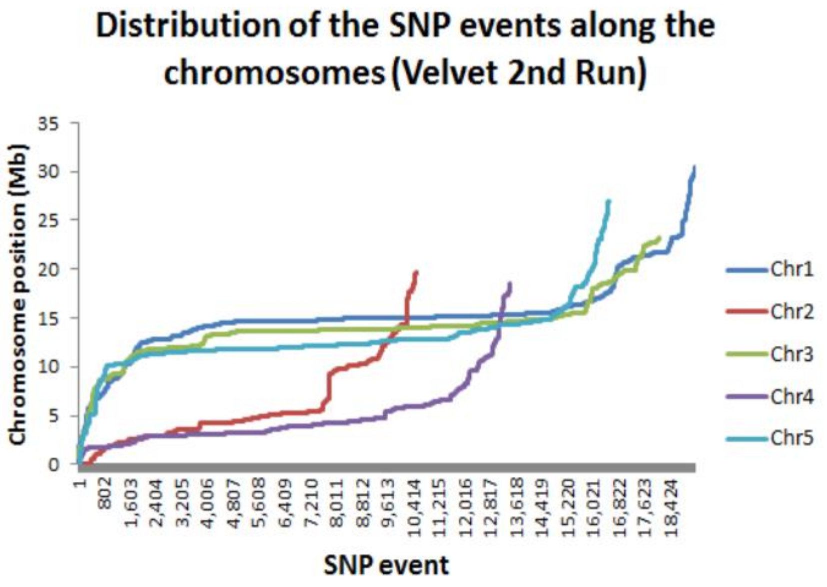

Fig. 12. Plot of SNP locations by chromosome, from the Velvet second replicate of the experiment. SNP events on the y axis are ordered by their position on the chromosome.

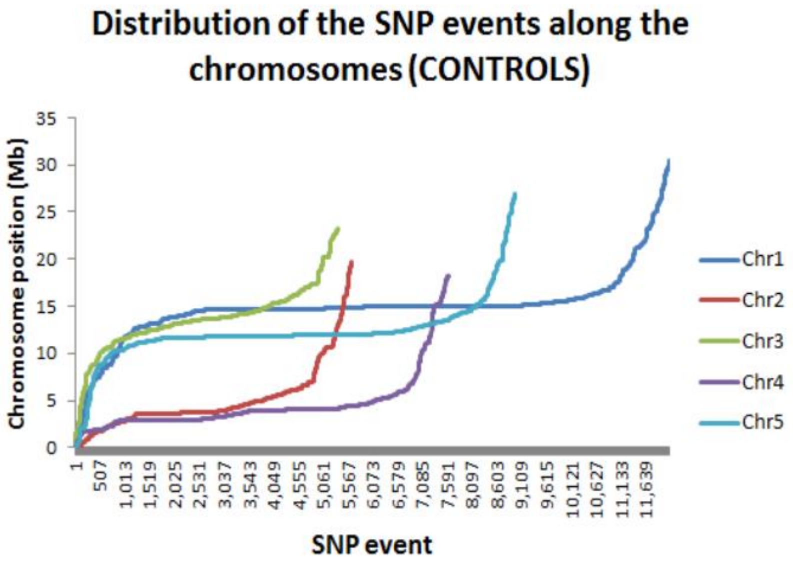

Fig. 13. Plot of SNP locations by chromosome, from the compiled controls of the experiment. SNP events on the y axis are ordered by their position on the chromosome.

SD.13 Source code and data availability

A short description of the file with the main source code used in this study is available at http://ics.hutton.ac.uk/software/tools/false-positive-snps.

The direct download link for the file is: http://ics.hutton.ac.uk/resources/antonio/fpSnpsCode.tar.gz.

The simulated reads used in this study are available for download from the following URL: https://ics.hutton.ac.uk/resources/antonio/reads/.

References

Earl, D., Bradnam, K., St. John, J., Darling, A., Lin, D., Fass, J., Paten, B. (2011). Assemblathon 1: A competitive assessment of de novo short read assembly methods. *Genome Research*, **21**(12), 2224–2241. doi:10.1101/gr.126599.111

Milne, I., Bayer, M., Cardle, L., Shaw, P., Stephen, G., Wright, F., Marshall, D.: Tablet – next generation sequence assembly visualization. *Bioinformatics*, **26**(3):401-402 (2010). doi: 10.1093/bioinformatics/btp666

Milne, I., Stephen, G., Bayer, M., Cock, P.J.A., Pritchard, L., Cardle, L., Shaw, P., Marshall, D.: Using Tablet for visual exploration of second-generation sequencing data. *Brief Bioinform*, **14**(2):193-202 (2013). doi: 10.1093/bib/bbs012

Cornish-Bowden, A.: Nomenclature for incompletely specified bases in nucleic acid sequences: recommendations 1984. Nucleic Acids Res. 1985;13:3021–3030
